# Genome-Wide Sequencing and an Open Reading Frame Analysis of Dichlorodiphenyltrichloroethane (DDT) Susceptible (*91-C*) and Resistant (*91-R*) *Drosophila melanogaster* Laboratory Populations

**DOI:** 10.1371/journal.pone.0098584

**Published:** 2014-06-10

**Authors:** Laura D. Steele, William M. Muir, Keon Mook Seong, M. Carmen Valero, Madhumitha Rangesa, Weilin Sun, John M. Clark, Brad Coates, Barry R. Pittendrigh

**Affiliations:** 1 Department of Entomology, University of Illinois, Urbana-Champaign, Urbana, Illinois, United States of America; 2 Department of Animal Sciences, Purdue University, West Lafayette, Indiana, United States of America; 3 Veterinary and Animal Sciences, University of Massachusetts, Amherst, Massachusetts, United States of America; 4 United States Department of Agriculture, Agricultural Research Service, Corn Insects & Crop Genetics Research Unit, Iowa State University, Ames, Iowa, United States of America; University of Crete, Greece

## Abstract

The *Drosophila melanogaster 91-R* and *91-C* strains are of common origin, however, *91-R* has been intensely selected for dichlorodiphenyltrichloroethane (DDT) resistance over six decades while *91-C* has been maintained as the non-selected control strain. These fly strains represent a unique genetic resource to understand the accumulation and fixation of mutations under laboratory conditions over decades of pesticide selection. Considerable research has been done to investigate the differential expression of genes associated with the highly DDT resistant strain *91-R*, however, with the advent of whole genome sequencing we can now begin to develop an in depth understanding of the genomic changes associated with this intense decades-long xenobiotic selection pressure. Here we present the first whole genome sequencing analysis of the *91-R* and *91-C* fly strains to identify genome-wide structural changes within the open reading frames. Between-strain changes in allele frequencies revealed a higher percent of new alleles going to fixation for the *91-R* strain, as compared to *91-C* (*P*<0.0001). These results suggest that resistance to DDT in the *91-R* laboratory strain could potentially be due primarily to new mutations, as well as being polygenic rather than the result of a few major mutations, two hypotheses that remain to be tested.

## Introduction

The organochlorine insecticide dichlorodiphenyltrichloroethane (DDT) disrupts arthropod nervous system function by affecting nerve cell plasma membrane permeability and causing paralysis [1]. The chemical was used for control of insect pest populations starting in the 1940s, but instances of field resistance were observed in many species including *Drosophila melanogaster* (*Drosophila*) [2]. Subsequent deleterious side effects arose in non-target mammalian and avian species that were linked to the environmental persistence of this insecticide [3], and contributed to usage bans in most countries during the modern environmental movement [4], [5]. However, DDT remains in industrial production due to its continued use for the control of malaria vectoring insects [6], and still persists in many ecosystems where it has been associated with negative effects on human health [7], [8], [9]. Despite the elimination of selection pressures in many nations, resistance traits persist within endemic pest populations and may remain at high frequencies due to random genetic drift on alleles that have no fitness cost [10]. Additionally, DDT resistance can confer cross-resistance to pyrethroid [11], [12] and neonicotinoid insecticides [13], and may be a factor that contributes to maintenance of resistance alleles in the absence of direct DDT selection [14]. However, ultimately, selection on insect populations through the use of DDT has been an important man-made evolutionary force.

*Drosophila* is a model for genomic research due to the existence of a high-quality genome sequence assembly, gene models and tools for genome-wide molecular analyses [9], [15]. With the advent of Next generation sequencing (NGS) technologies, full-genome re-sequencing has become logistically feasible, and allows for ultra-fine resolution in mutation mapping [16] and genome-wide association studies (GWAS), population genomics [17] and phylogenomics [18]. GWAS, based on NGS approaches, has been effective in the identification of genome regions that influence the expression of traits in crop plants [19], [20], [21]. The laboratory selected DDT resistance traits in *Drosophila* strain *91-R* has been under differential selection pressures for>60 years while *91-C* provides a non-selected control strain that retains its DDT susceptibility; the two strains came from a common population that was split before these decades-long difference in treatment of the two populations [22], [23], [24].

Interestingly, this selection parallels another scenario in wild mice and rats, where decades long selection pressure has resulted in novel mutations that confer warfin resistance [25]. However, the work by Rost et al. [25] focused on the vitamin K reductase (VKOR) gene, an important target of warfin – not on a whole genome analysis. Beyond insecticide resistance, the *91-R* and *91-C* strains represent a unique opportunity to determine how such intense selection pressure has impacted a genome as compared to it’s non-selected counter-part. For example, at the current moment we do not know if decades long DDT selective pressure primarily results in fixation of old, standing variation alleles or new allelic variants and are these novel mutations restricted to genes known to be associated with resistance or are there novel open reading frame mutations (ORFs) across the genome that have also gone to fixation. Further, the number of genes involved in the evolution of resistance is unknown and could be the result of a few macro-mutations with large effects, or the cumulative effects of many polygenic loci each with small effects.

Here we performed whole genome re-sequencing and single nucleotide polymorphism (SNP) analysis and deletion/insertion polymorphisms (DIP) analysis of the *Drosophila* strains *91-R* and *91-C* to detect types of mutations putatively affecting the evolution of DDT resistance, both in terms of standing *vs.* new mutations, and the amino acid changes in the ORFs of known genes.

## Results

### Genome Re-sequencing, Read Mapping, and Detection of Single Nucleotide Polymorphisms

To test the hypothesis that selection in line *91-R* opportunistically utilized new mutations as it evolved DDT resistance, we mapped the sequencing data from *91-R* and *91-C* to the *Drosophila simulans* reference genome. *D. simulans* is the closest out group to *Drosophila*, hence any alleles in common with the *D. simulans* reference are old mutations and may have existed prior to the divergence of these two species. Thus in terms of the allele frequency distribution, we were able to unfold the distribution rather than working with minor allele frequency (MAF), i.e. ancestral alleles found in *D. melanogaster* will be similar to the *D. simulans* reference while new mutations will be dissimilar and usually only found in the *D. melanogaster* population. Stated in another way, when the *D. simulans* reference was used, alleles that were similar to *D. simulans* have values close to 0, but also represent alleles that existed prior to the divergence of the species, and were classified as old alleles. Similarly, those that were dissimilar to *D. simulans* have values close to 1.0, and primarily represent alleles that arose after these species diverged and thus were classified as new alleles.

Fastq data from the Illumina Genome Analyzer IIx (GAIIx) contained 139,299,388 and 148,106,954 paired end reads for strains *91-C* and *91-R* respectively, and post-processing reduced the number of reads to 104,420,681 and 107,365,941, respectively. Mapping of the trimmed *91-C* and *91-R* read data to the *D. simulans* reference genomes respectively resulted mean coverage depths of 55.6 and 56.5 and to the *Drosophila* reference genomes mean coverage depths of 64.9 - and 63.1-X (Table 1).

**Table 1:** Summary of the mapping of reads to the reference *Drosophila melanogaster* genome* and calling of SNPs/DIPs causing animo acid changes.

	*91-C* Fly line	*91-R* Fly line
Total number ofIllumina reads	139,299,388	148,106,954
Number of mapped reads	104,420,681	107,365,941
Average coverage	31.8X	31.0X
Number of SNPs/DIPscausing amino acid changes	779	710

*Drosophila melanogaster* genome release 5.36 from flybase.org.

In total we found 2,407,685 SNPs against the *D. simulans* reference genome. Of those alleles, 1,124,747 were fixed and in common to both lines, 663,066 were unique to *91-R* while only 619,872 were unique to *91-C*. The difference in unique alleles between lines was 43,194 SNPs and highly significant (*P*<0.0001). Thus 1.79% of all new alleles (mutations) were associated with differentiating *91-R* from *91-C* and at least some are putatively related to DDT resistance. Although random genetic drift can fixate different alleles in the lines, with neutral alleles the probability of fixating a new allele in either line is the same. And as seen, the majority of alleles (98.2%) that are different between lines are due to mutations driven by random genetic drift; on average there was a 2.6% excess of alleles associated with the DDT selected line and at least some may be putatively associated with DDT resistance.

**Figure 1:**
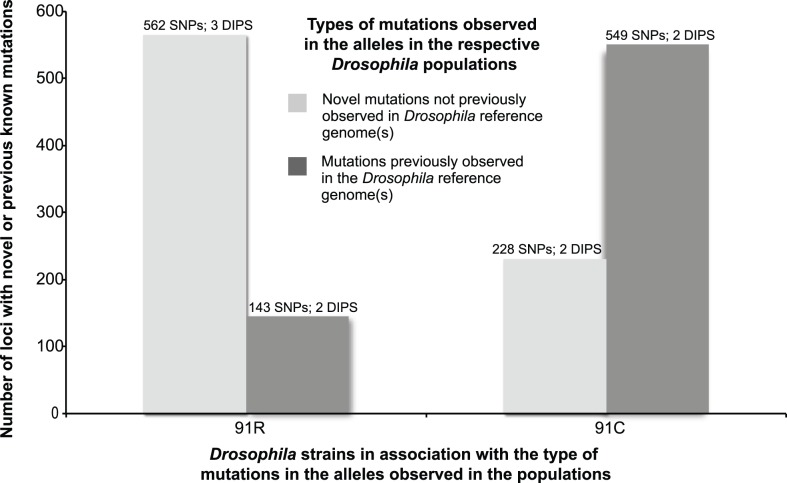
A comparison of allele fixation for the *Drosophila melanogaster* (*Drosophila*) fly lines 91-R and 91-C, in their respective populations, in relation to novel and previously known mutations. The figure represents only those mutations observed in the *91-R* and *91-C* genomes that caused amino acid structural changes in open reading frames (ORFs) resulting from a single nucleotide polymorphisms (SNPs) or deletion/insertion polymorphisms (DIPs). A graphical representation of allele fixation for both *91-R* and *91-C* across the entire *Drosophila* genome from each of the two respective populations.

**Figure 2:**
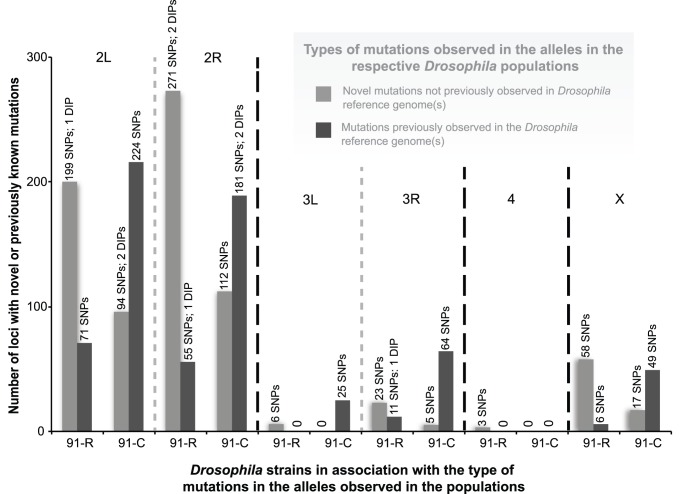
A comparison of allele fixation for the *Drosophila melanogaster* (*Drosophila*) fly lines *91-R* and *91-C*, in their respective populations, in relation to novel and previously known mutations. The figure represents only those mutations observed in the *91-R* and *91-C* genomes that caused amino acid structural changes in open reading frames (ORFs) resulting from a single nucleotide polymorphisms (SNPs) or deletion/insertion polymorphisms (DIPs). Observed allele fixation events separated into the individual *Drosophila* chromosomes (4 and X) or by chromosomal arms (2R, 2L, 3R, and 3L).

Comparison of polymorphism among reads from *91-C* and *91-R* to the *Drosophila* reference genome assembly (release 5.36) were used to predict the frequency of segregating SNPs and the position of fixed nucleotide differences within ORFs. These mutations were categorized according to derived or shared between strains *91-C* and *91-R* (Table 2). In terms of amino acid changes in ORFs, intra-strain polymorphisms were detected with both *Drosophila* strains, where a total of 781 SNPs and DIPs were predicted among reads from *91-C* and 710 SNPs and DIPs were found among reads from strain *91-R* (Figure 1). The distribution of the types of mutation (novel or reference) was dependent on the specific fly strain (χ^2^ = 375.47, df = 1, *P*<0.01) (Figure 1). These results indicated that the number of genome positions within the *91-R* strain that represent novel mutations were higher compared to strain *91-C*. Specifically, a total of 562 SNPs and three DIPs loci from *91-R* were fixed for different alleles when compared to the reference. In contrast, strain *91-C* shared a greater proportion of nucleotide similarity with the *Drosophila* reference genome (i.e. lower number of novel alleles; 228 SNPs and two DIPs that went to fixation for novel alleles). Of the 562 genome positions that were fixed differently within strain *91-R* and the reference genome, 549 SNPs and three DIPs were fixed for the novel SNP/DIP alleles not previously published in the reference alleles (Table 3; Figure 1). Two of these novel nucleotide changes within strain *91-R* (Figure 1) were located within the Arm U (Table 2), but were excluded from further analyses (Figure 2).

**Table 2:** Number of genes with one or more SNP(s) and DIP(s) (causing an amino acid change) located within the open reading frame for the *91-R* and *91-C* fly lines.

Chromosome Arm	*91-R* Only		*91-R* & *91-C*		*91-C* Only	
	TotalNumberof SNPs & DIPs	Number of Geneswith SNPs & DIPs	Total Number ofSNPs & DIPs	Number of Genes withSNPs & DIPs	Total Number ofSNPs & DIPs	Number of Geneswith SNPs & DIPs
2L	136	42	327	59	130	51
2R	198	78	278	51	161	75
3L	6	1	0	0	25	4
3R	23	4	39	6	43	23
4	3	2	0	0	0	0
X	37	11	52	11	46	18
U	2	0	0	0	0	0
Totals	405	138	696	127	405	171

**Table 3:** Comparison of the total number of genes on each chromosome arm and the number of genes with SNPs/DIPs† (causing an amino acid change) located within the open reading frame for the *91-R* and *91-C* fly lines.

Chromosome Arm	Number of Genes*	*91-R* Only	*91-R* & *91-C*	*91-C*
2L+2Lhet	2939	1.4%	2.0%	1.7%
2R+2Rhet	3238	2.4%	1.6%	2.3%
3L+3Lhet	2950	0.0003%	0%	0.14%
3R+3Rhet	3702	0.12%	0.16%	0.62%
4	90	2.2%	0%	0%
X	2381	0.46%	0.46%	0.76%

*Retrieved from Flybase.org (FlyBase Release FB2012_02, *D. melanogaster* annotation Rel_5.44).

† Refer to Table 3 for these values.

### Separation of SNPs/DIPs, and Genes Containing SNPs/DIPs, by Individual Chromosome

An examination of the SNPs/DIPs across each individual chromosome identified a specific number of mutations per chromosome and in turn, the specific genes containing one or more mutations. This analysis was performed separately for (1) the genes containing SNPs/DIPs from the *91-R* fly strain, (2) the genes containing SNPs/DIPs from the *91-C* fly strain, and (3) the genes containing SNPs/DIPs from both the *91-R* and *91-C* fly strains (Table 2). Based on this analysis, it appears that there are a higher number of SNPs/DIPs, as well as a higher number of genes containing mutations, found on the second chromosome across all three defined categories.

To elaborate beyond solely looking at the numbers of genes containing mutations, a comparison of the total number of genes containing one or more SNP/DIP to the total number of genes on each chromosome (or chromosome arm) of the *Drosophila* genome provided percentages giving an overall idea of the distribution of the structural changes across the genome (Table 3). The analysis was once again separated for those genes containing SNPs/DIPs from the *91-R* fly strain, the genes containing SNPs/DIPs from the *91-C* fly strain, and the genes containing SNPs/DIPs from both the *91-R* and *91-C* fly strains, but interestingly, all three categories consistently had the highest percentage of changes on the second chromosome. The SNPs/DIPs for the *91-R* only also showed a high percentage of genes with mutations occurring on the fourth chromosome, though the other two categories had no mutations appearing in any of the genes on the fourth chromosome.

### SNP/DIP Identification and their Molecular/Biological Functions

**Figure 3:**
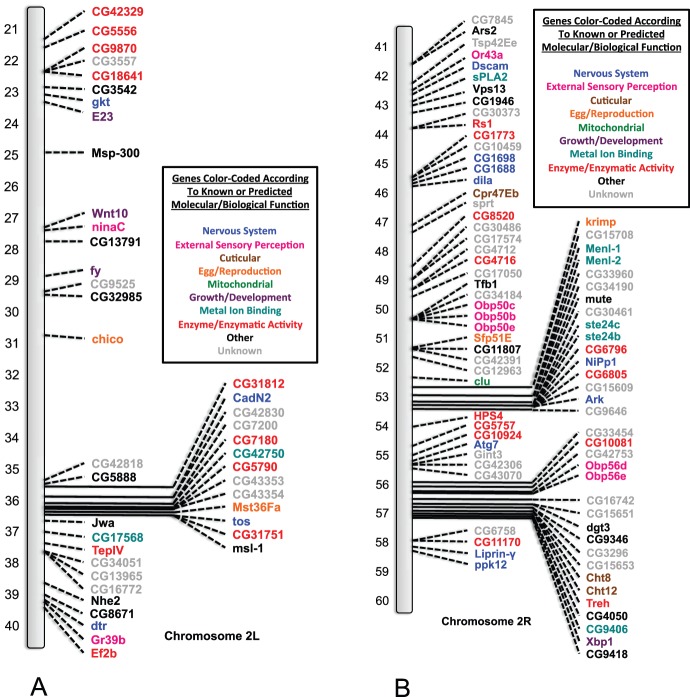
Genes containing mutations identified on the left and right arm of the second chromosome (respectively 2L and 2R) in the *91-R* strain only. *Drosophila melanogaster* (*Drosophila*) chromosomes containing the cytogenetic map locations of those genes identified as containing one or more single nucleotide polymorphisms (SNPs) or deletion/insertion polymorphisms (DIPs) that caused an amino acid changes in open reading frames (ORFs) in the *91-R* strain only. Genes are labeled by their corresponding symbols, as provided on flybase.org, and color-coded according to their known or predicted molecular/biological function (from uniprot.org or other literature sources). Where applicable, other molecular/biological functions for these genes are given in Table S1, along with additional literature sources. The categories given are: (1) nervous system, (2) external sensory perception, (3) cuticular, (4) egg/reproduction, (5) mitochondrial, (6) growth/development, (7) metal ion binding, (8) enzyme/enzymatic activity, (9) other and (10) unknown. The chromosome is represented by the grey bar and the cytogenetic map reference locations are given for the left arm of the second chromosome: 2L (21–40) (Figure 3A) and right arm of the second chromosome: 2R (41–60) (Figure 3B).

**Figure 4:**
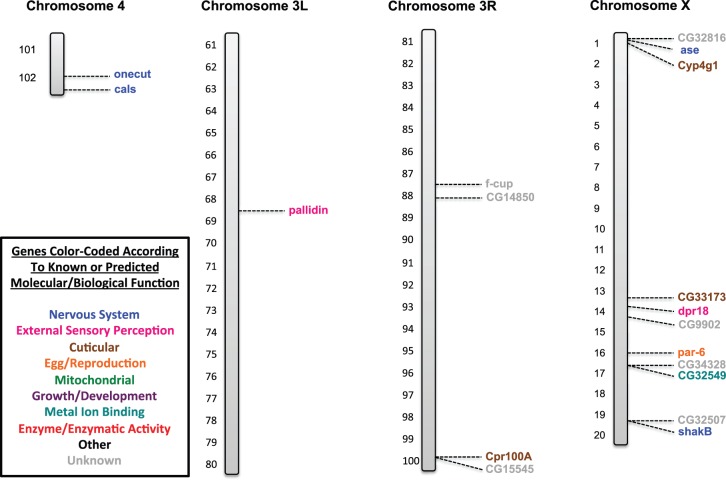
Genes containing mutations identified on the fourth chromosome (4), on the left arm of the third chromosome (3L), on the right arm of the third chromosome (3R), and on the X chromosome in the *91-R* strain only. *Drosophila melanogaster* (*Drosophila*) chromosomes containing the cytogenetic map locations of those genes identified as containing one or more single nucleotide polymorphisms (SNPs) or deletion/insertion polymorphisms (DIPs) that caused an amino acid changes in open reading frames (ORFs) in the *91-R* strain only. Genes are labeled by their corresponding symbols, as provided on flybase.org, and color-coded according to their known or predicted molecular/biological function (from uniprot.org or other literature sources). Where applicable, other molecular/biological functions for these genes are given in Table S1, along with additional literature sources. The categories given are: (1) nervous system, (2) external sensory perception, (3) cuticular, (4) egg/reproduction, (5) mitochondrial, (6) growth/development, (7) metal ion binding, (8) enzyme/enzymatic activity, (9) other and (10) unknown. The chromosomes are represented by the grey bars and the cytogenetic map reference locations are given for each of the chromosomes: X (1–20), 3L (61–80), 3R (81–100), and 4 (101–102).

**Figure 5:**
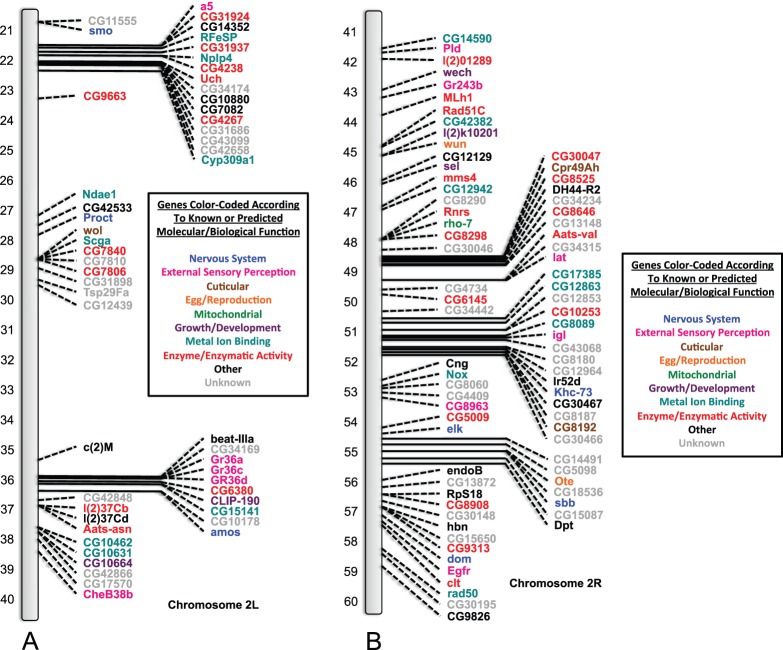
Genes containing mutations identified on the left and right arm of the second chromosome (respectively 2L and 2R) in the *91-C* strain only. *Drosophila melanogaster* (*Drosophila*) chromosomes containing the cytogenetic map locations of those genes identified as containing one or more single nucleotide polymorphisms (SNPs) or deletion/insertion polymorphisms (DIPs) strain that caused an amino acid change in the *91-C*. Genes are labeled by their corresponding symbols, as provided on flybase.org, and color-coded according to their known or predicted molecular/biological function (from uniprot.org or other literature sources). Where applicable, other molecular/biological functions for these genes are given in Table S2 along with additional literature sources. The categories given are: (1) nervous system, (2) external sensory perception, (3) cuticular, (4) egg/reproduction, (5) mitochondrial, (6) growth/development, (7) metal ion binding, (8) enzyme/enzymatic activity, (9) other and (10) unknown. The chromosome is represented by the grey bar and the cytogenetic map reference locations are given for the left arm of the second chromosome: 2L (21–40) (Figure 5A) and right arm of the second chromosome: 2R (41–60) (Figure 5B).

**Figure 6:**
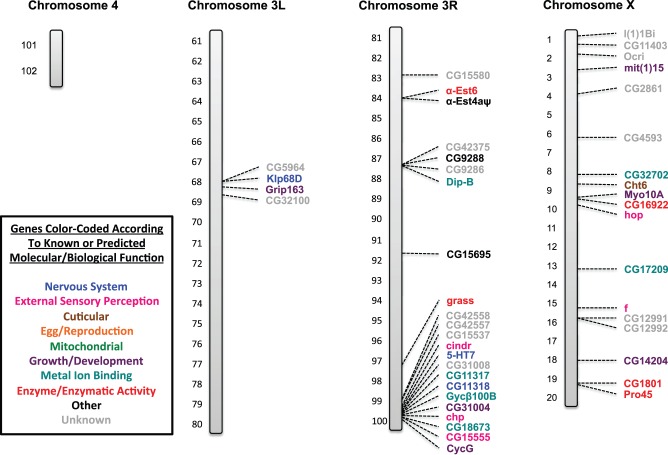
Genes containing mutations identified on the fourth chromosome (4), on the left arm of the third chromosome (3L), on the right arm of the third chromosome (3R), and on the X chromosome in the *91-C* strain only. *Drosophila melanogaster* (*Drosophila*) chromosomes containing the cytogenetic map locations of those genes identified as containing one or more single nucleotide polymorphisms (SNPs) or deletion/insertion polymorphisms (DIPs) strain that caused an amino acid change in the *91-C*. Genes are labeled by their corresponding symbols, as provided on flybase.org, and color-coded according to their known or predicted molecular/biological function (from uniprot.org or other literature sources). Where applicable, other molecular/biological functions for these genes are given in Table S2, along with additional literature sources. The categories given are: (1) nervous system, (2) external sensory perception, (3) cuticular, (4) egg/reproduction, (5) mitochondrial, (6) growth/development, (7) metal ion binding, (8) enzyme/enzymatic activity, (9) other and (10) unknown. The chromosomes are represented by the grey bars and the cytogenetic map reference locations are given for each of the chromosomes: X (1–20), 3L (61–80), 3R (81–100), and 4 (101–102).

Those genes containing at least one or more of the SNPs and/or DIPs for both the *91-R* and *91-C* fly strains were identified. It should be noted once again that the identified mutations were separated into three categories: (1) genes that contained only SNPs and DIPs from the *91-R* resistant fly strain; (2) genes that contained SNPs and DIPs from both the *91-R* resistant fly strain and the *91-C* susceptible strain; and (3) genes that contained only SNPs and DIPs from the *91-C* susceptible fly strain (Table 2). Genes associated with multiple molecular/biological functions had their relevant function assigned into one of the following categories (1) nervous system, (2) external sensory perception, (3) cuticular, (4) egg/reproduction, (5) mitochondrial, (6) growth/development, (7) metal ion binding, (8) enzyme/enzymatic activity, (9) other and (10) unknown (Table S1). Each gene was mapped on the *Drosophila* chromosome figures at its cytogenetic map location using its corresponding symbol annotation from flybase.org and color-coded according to the molecular/biological functions as listed previously (Figures 3, 4, 5, 6). Genes from all three categories were highlighted initially and further analysis was focused solely on those SNPs and DIPs from the *91-R* resistant strain with the objective to isolate potential structural changes that may confer or be related to insecticide resistance.

## Discussion

To the best of our knowledge, this study represents the first WGS analysis of a highly pesticide resistance and susceptible strains, of common origin, in order to determine the presence of known or unique allelic variants. The Illumina sequencing of the DDT susceptible *91-C* and DDT resistant *91-R Drosophila* fly strains and subsequent analysis allowed us (i) to compare the DDT resistant and susceptible populations for differences and similarities across ORFs in the whole genome, (ii) as well as through gene by gene examination of individual nucleotide changes causing amino acid differences, and (iii) potential structural changes within the ORFs. The fly strain under pesticide selective pressure, *91-R*, had a higher number of novel alleles that had gone to fixation as compared to the DDT susceptible *91-C* fly strain. Conversely, the *91-C* fly strain had a higher number of alleles that had gone to fixation which were not novel (Figure 1). These identical patterns of allele fixation were observed across all of the individual chromosomes, except for the fourth chromosome (Figure 2). Cytogenetic mapping of the genes containing one or more SNP/DIP resulted in visual clusters of genes on specific chromosomes, which could potentially indicate selection history specific to these two strains and could be a direction for future study. Further SNP/DIP analysis for those mutations from only the *91-R* strain identified 138 genes across a range of molecular/biological functions that could be potential candidate DDT resistance genes (Figure 3A&B Figure 4). When looking at the percentage of genes containing mutations on each *Drosophila* chromosome arm for only the *91-R* fly strain, there were higher percentages found on the right arm of the second chromosome and the fourth chromosome, followed by the left arm of the second chromosome.

Moderate to high level DDT resistance is thought to be polygenic with multiple chromosomes contributing to this phenotype. A number of articles identified the second chromosome, and even specific loci on this chromosome [26], [27] to be involved in DDT resistance. Chromosomes one and three are thought to have some slight impact on the DDT resistant phenotype [26], [28]. An examination of the results for this WGS analysis appears to follow these early chromosomal studies on DDT resistance, at least based on the locations of the genes with mutations. The majority of the genes with *91-R* only SNPs/DIPs were positioned on the second chromosome, with some additional genes on the remaining three chromosomes (Table 2). This was further confirmed by normalizing the number of genes with mutations with the total number of genes per *Drosophila* chromosome arm to calculate overall percentages of change (Table 3). Our analysis, in addition to the *91-R* strain, found the *91-C* strain to also have a higher number of SNPs/DIPs located on the second chromosome (Figure 5A&B Figure 6). This could potentially be due to unintentional laboratory selection of this population by exposure to xenobiotic compounds, such as in the diet or environment, as the characterization of P450s, xenobiotic/detoxification enzymes, as over half of the P450s identified in *Drosophila* were located on the second chromosome. It should also be noted that some, or all, of the changes located in both the *91-R* and *91-C* strains could have been previously found in their common population of origin (Figures S1, S2, and S3) [29].

Our WGS analysis suggests that selection for resistance, at least in the case of *91-C* and *91-R*, may also result in a differential type of mutations going to fixation in the genome – with mainly novel mutations being associated with intense pesticide selection over numerous generations, a hypothesis that would need to be further tested with other pairs of susceptible and resistant strains of common origin. The current work adds one more critical aspect to our understanding of the molecular changes that have occurred under DDT selection as previous work respectively defined genome-wide and partially proteome-wide differential expression of transcripts and proteins [30], [31]. With a total number of resistant SNPs/DIPs of 710, and 565 of these being novel mutations, spanning across all four chromosomes, it highlights that selection with DDT may result, on average, with the selection for novel mutations, potentially with some or many of these being associated with or directly involved in DDT resistance (Figure 2). It will be important to do follow-up analyses on the individual genes to verify the mutations, thus confirming their structural changes and potential impact on the resistance phenotype of *91-R*. Further molecular examination of individual genes and a more detailed analysis of the specific effects of the structural changes within the insects are crucial to better understanding resistance, something beyond the scope of the current project.

In contrast to previous “omics” scale analyses of DDT resistant *Drosophila* strains, the current work focuses on the structural changes within ORFs in the *Drosophila* genome that have occurred in a highly selected strain as compared to a non-selected strain, both of common origin (Table 4). A search of current and past literature was completed to survey previously known/hypothesized DDT resistance genes identified from *Drosophila,* encompassing both differentially expressed genes and genes containing structural changes. The literature, to date, as focused on differential expression or over-expression of genes when *Drosophila* strains were exposed to DDT revealed a set of cytochrome P450s, a set of Glutahione S Transferases, and a set additional genes, three of which were still significant with a Bonferroni correction (Table 4). The P450 gene *CYP6G1* was recognized by all nine references as playing a role in resistance, with five references specifically indicating that it is thought to confer resistance. In addition to *CYP6G1*, Festucci-Buselli *et al.* [32] identified *CYP12D1* as conferring resistance to DDT. The only gene identified in the literature search that overlapped with this study was *Nina C*, a gene with kinase activity and related to sensory transduction/vision, which was shown by Pedra *et al.* [31] to be overexpressed in DDT resistant strains and shown in this study to contain structural changes. Literature examining structural changes revealed two genes, *CYP6A2* and *para*, to contain mutations within their coding sequence (Table 5). Interestingly, there was almost no overlap between the genes with observed SNPs/DIPs and the genes that have been previously associated with DDT resistance in pesticide resistant *Drosophila* populations. However, our current study was not focused on pesticide resistance, but instead on the impact of DDT selection on the presence of unique versus previously known alleles on the insect population.

**Table 4:** Differential expression or over-expression of genes when *Drosophila melanogaster* strains are exposed to DDT (often comparisons between resistant and susceptible *Drosophila* strains).

Gene Detoxification Enzyme Categories				
Cytochrome P450s	Glutathione S Transferases	Esterases	Other	Citation
*CYP12D1*, *CYP6G1*, *CYP6A2*	GSTE8, GSTE3, GSTD2, GSTD1,CG6781, GSTE9, GSTE6,GSTE5, GSTE1, CG1702,CG16936, GSTE3, GSTD2,CG1681, GSTE9, GSTE5, GSTD4			Sun *et al*., 2011
*CYP6G1* *, *CYP12D1*				Daborn *et al.*, 2007
*CYP6G1* *, *CYP12D1* *				Festucci-Buselli *et al.*, 2005
*CYP6A2*, *CYP12D1*			Dbi, Uhg1, CG11176	Pedra *et al., 2004*
*Genes that cut past Bonferonni correction, as described in Pedra et al., 2004.*				
*CYP6A2*, *CYP12D1*, *CYP6A17*,*CYP6A8*, *CYP12D1*, *CYP6W1*,*CYP6G1*, *CYP6A14*, *CYP9C1*,*CYP4P1*, *CYP6A23*	CG17530, CG17522, CG1681,Gst3-1, CG6673		Ugt86Dh, Ugt86Dd, Ugt35b, CG541, Pdh,CG30019, CG3301, CG12224, CG8888,CG9360, CG3603, CG3842, CG15531,CG9747, CG15093, InaF, CG17142,Cpn, CG2185, Ca-α1D, Mys, CG16727,Rya-r44F, CG8932, CG15407, CG9362,CG5568, CG5397, CG17192, CG9510,Yip2, CG10737, Ext2, Dbi, CG14715,CG9892, Arr1 Arr2, Dia, Ank2,Map205, Sox100B, Cf2, NFAT,Odd, Nut2, CG3091, Lectin-galC1,CG11211, Rh4, Rh3, Glob1, Nina E,Nina C, CG10355, CG7409.CG10467, CG8505, CG1304,CG10477, CG11034, CG9897,Ser12, BG:BACR44L 22,EG:100G10.4, InaC,Ggamma30A, Gbeta76C, Sr-Cl,Or92a, LysD, LysB,LysC, LysE, LysP,l(2)06225, InaD, Qm, Pi3K59F	
*Genes that cut past P<0.01, as described in Pedra et al., 2004.*				
*CYP6G1*, *CYP6A8*, *CYP12D1*/*CYP12D2*				Le Goff *et al.*, 2003
*CYP6G1* *, *CYP12D1*				Brandt *et al.*, 2002
*CYP6G1* *				Daborn *et al.*, 2002
*CYP6G1* *				Daborn *et al.*, 2001

*indicates that gene is thought to confer DDT resistance (as indicated specifically by the cited article).

**Table 5:** Structural changes of genes when *Drosophila melanogaster* strains are exposed to DDT.

Gene Category	Gene Name	Structural Change Description	Citation
Cytochrome P450	*CYP6A2*	3 Point Mutations(R335S, L336V, V476L)	Amichot *et al.*, 2004
Voltage-gatedsodium channel	*para*	Mutation within intracellular loopbetween S4 and S5 (homology domains I and II).Mutation within pore region (homology domain III).Mutation within S6 (homology domain III).Mutations first isolated by temperature sensitive bioassay and thensubsequently assessed for DDT resistance.	Pittendrigh *et al.*, 1997

The WGS of the *91-C* and *91-R* fly strains may ultimately provide an example of a method to investigate the whole genome impact of insecticide selection on pest populations. Certainly, this general WGS and genomic structural change analysis approach could be applied to these other insect species currently being controlled by other pesticides, in order to understand genome-wide evolution of population of insects being exposed to pesticides in “real time” (i.e. follow field populations through generations of selection). As the number of other insect genomes have been sequenced since the *Drosophila* genome was published in 2000, it has allowed for the study of insecticide resistance at the molecular level for a variety of species, such as in *Anopheles gambiae* [15], [33]. Although the *91-C* and *91-R* fly strains provide a unique system where selection has occurred for over half a century, there exist multiple *Aedes aegypti* laboratory strains, including strains selected for insecticide resistance to permethrin, where similar studies could be performed to identify structural mutations across the genome [34]. The discoveries of novel resistance mechanisms from such studies could help lead to genomic changes that occur in target species under selection pressure by pesticide treatments [35], [36].

## Materials and Methods

### Genome Re-sequencing, Read Mapping, and Detection of Single Nucleotide Polymorphisms

*Drosophila* strains *91-R* and *91-C* were obtained from Dr. Ranjan Ganguly (University of Tennessee-Knoxville), and developed as described by Merrell and Underhill [22]. Strains were reared on brown diet (Jazz-Mix Drosophila Food, Fischer Scientific, Cat. No. AS153) at ∼25°C 8∶16 L∶D in plastic bottles and transferred to new bottles about every three weeks. DDT resistance was verified in the *91-R* fly strain and susceptibility in the *91-C* by conducting a DDT-exposure bioassay (at DDT concentrations of 0, 10, 20, and 100 µg/ml). A total of 1000 male and 1000 female flies were collected from each strain, flash froze in liquid nitrogen, and stored at −80°C. Male and female flies from each respective fly strain were pooled and then split into random pools of ∼1000 flies (males and females mixed). DNA was extracted from these mixed pools (males and females) of flies using the Qiagen DNeasy Plant Maxi Kit (Qiagen, Valencia, CA; Cat. No. 68163, Lot. No. 430117979) according to the manufacturer’s instructions. DNA samples were quantified using NanoDrop 1000 UV/VIS Spectrophotometer (Thermo Scientific, Serial No. G642) and quality was estimated by 0.9% agarose gel electrophoresis. Two 300 bp paired-end insert libraries were constructed and 100 bp paired end reads were generated on an Illumina Genome Analyzer IIx (GAIIx) at the W.M. Keck Center for Comparative and Functional Genomics at the University of Illinois Urbana-Champaign with two lanes run for each *91-R* and *91-C*. Bases were called using Illumina software and data outputted as.fastq files.

The fastq formatted files of paired end Illumina GAIIx data were input into CLC Genomics Workbench 5.1 software, and aligned to the reference *Drosophila* genome (version 5.3.6; File: dmel-all-chromosome-r5.36.fasta.gz at ftp://ftp.flybase.net/genomes/Drosophila_melanogaster/) using the local alignment option. The range of paired-end reads limited to 200 to 430bp. SNPs and DIPs were called based on CLC Genomics Workbench 5.1 SNP calling software with the requirements that the central SNP/DIP have a PHRED quality (*q*) score ≥20 and the11 flanking nucleotides on either side have a *q≥*15, a coverage depth *≥*10 and a variant nucleotide frequency ≥0.10. Illumina GAIIx reads were submitted to the National Center for Biotechnology Information (NCBI) sequence read archive (SRA) under the data accession number SRP041176.

### Genome-wide Signatures of DDT Selection

Estimates of the *F_ST_* statistic were generated and used as a measure of genomic DNA sequence variation among strains, and high estimates interpreted as genome regions that have significant sequence divergence (potentially due to a local selective sweep caused by DDT selection within *91-R*). In brief, *F_ST_* estimates were calculated from population heterozygosities within a 10,000 bp sliding window using custom Fortran 95 scripts with the equations given below (Table 6). Additionally, the nucleotide diversities (equation) was estimated between *91-R* and *91-C* using within a 10,000 bp sliding window and calculated in 1,000 bp steps across the genome. Nucleotide diversity indices were used to detect genome regions with high inter-strain diversity, and interpreted as genome regions that may show differential nucleotide fixation due to the effects of DDT selection.

**Table 6:** Equations utilized to calculate *F_ST_* estimates from population heterozygosities within a 10,000 bp sliding window.

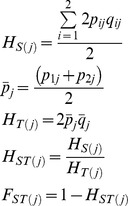	and over a sliding window with N SNPs	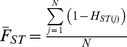

The allele frequency at locus *j* in population *i* is *p_ij_*. Estimates of F_ST_>.8 across a given sliding window was considered extreme.

The genes that contained ≥1 novel fixed SNPs and/or DIPs among strains *91-R* and *91-C* when compared to the *Drosophila* WGS release 5.36 were identified, and then the full sequence ORF were extracted from the gene models (dmel-all-chromosome-r5.36.fasta.gz) and grouped into a multi-FASTA file (Dm_SNPcds.fasta). Processed reads from *91-C*, *91-R* and those assembled within release 5.36 were mapped independently to sequences within Dm_SNPcds.fasta, and consensus sequences output into individual FASTA formatted files.

## Supporting Information

Figure S1 **Genes containing mutations were identified on the left arm of the second chromosome (2L) in both the** ***91-R and 91-C*** **strains.** *Drosophila melanogaster* (*Drosophila*) chromosomes containing the cytogenetic map locations of those genes identified as containing one or more single nucleotide polymorphisms (SNPs) or deletion/insertion polymorphisms (DIPs) strain that caused an amino acid change in both the *91-R* and *91-C* strains. Genes are labeled by their corresponding symbols, as provided on flybase.org, and color-coded according to their known or predicted molecular/biological function (from uniprot.org or other literature sources). Where applicable, other molecular/biological functions for these genes are given in Table S3, along with additional literature sources. The categories given are: (1) nervous system, (2) external sensory perception, (3) cuticular, (4) egg/reproduction, (5) mitochondrial, (6) growth/development, (7) metal ion binding, (8) enzyme/enzymatic activity, (9) other and (10) unknown. The grey bar represents the chromosome and the cytogenetic map reference locations are given for the left arm of the second chromosome: 2L (21–40).
(EPS)

Figure S2 **Genes containing mutations were identified on the right arm of the second chromosome (2R) in both the** ***91-R and 91-C*** **strains.** *Drosophila melanogaster* (*Drosophila*) chromosomes containing the cytogenetic map locations of those genes identified as containing one or more single nucleotide polymorphisms (SNPs) or deletion/insertion polymorphisms (DIPs) strain that caused an amino acid change in both the *91-R* and *91-C* strains. Genes are labeled by their corresponding symbols, as provided on flybase.org, and color-coded according to their known or predicted molecular/biological function (from uniprot.org or other literature sources). Where applicable, other molecular/biological functions for these genes are given in Table S3, along with additional literature sources. The categories given are: (1) nervous system, (2) external sensory perception, (3) cuticular, (4) egg/reproduction, (5) mitochondrial, (6) growth/development, (7) metal ion binding, (8) enzyme/enzymatic activity, (9) other and (10) unknown. The grey bar represents the chromosome and the cytogenetic map reference locations are given for the right arm of the second chromosome: 2R (41–60).
(EPS)

Figure S3 **Genes containing mutations identified on the fourth chromosome (4), on the left arm of the third chromosome (3L), on the right arm of the third chromosome (3R), and on the X chromosome in both the** ***91-R*** **and** ***91-C*** **strains.** *Drosophila melanogaster* (*Drosophila*) chromosomes containing the cytogenetic map locations of those genes identified as containing one or more single nucleotide polymorphisms (SNPs) or deletion/insertion polymorphisms (DIPs) strain that caused an amino acid change in both the *91-R* and *91-C* strains. Genes are labeled by their corresponding symbols, as provided on flybase.org, and color-coded according to their known or predicted molecular/biological function (from uniprot.org or other literature sources). Where applicable, other molecular/biological functions for these genes are given in Table S3, along with additional literature sources. The categories given are: (1) nervous system, (2) external sensory perception, (3) cuticular, (4) egg/reproduction, (5) mitochondrial, (6) growth/development, (7) metal ion binding, (8) enzyme/enzymatic activity, (9) other and (10) unknown. The grey bars represent the chromosomes and the cytogenetic map reference locations are given for each of the chromosomes: X (1–20), 3L (61–80), 3R (81–100), and 4 (101–102).
(EPS)

Table S1 **Molecular and biological functions, obtained from uniprot.org and literature searches, for those genes containing SNPs/DIPs from only the** ***91-R*** **fly line. Gene symbol, gene name, and annotation symbol from flybase.org.** The color-coding system is as follows: Nervous system = blue, External sensory perception = pink, Cuticular = brown, Egg/Reproduction = orange, Mitochondrial = green, Growth/Development = purple, Metal ion binding = teal, Enzyme/Enzymatic activity = red, Other = white, Unknown = gray.
(DOCX)

Table S2 **Molecular and biological functions, obtained from uniprot.org and literature searches, for those genes containing SNPs/DIPs from only the** ***91-C*** **fly line. Gene symbol, gene name, and annotation symbol from flybase.org.** The color-coding system is as follows: Nervous system = blue, External sensory perception = pink, Cuticular = brown, Egg/Reproduction = orange, Mitochondrial = green, Growth/Development = purple, Metal ion binding = teal, Enzyme/Enzymatic activity = red, Other = white, Unknown = gray.
(DOCX)

Table S3 **Molecular and biological functions, obtained from uniprot.org and literature searches, for those genes containing SNPs/DIPs from both the** ***91-C*** **and** ***91-R*** **fly line.** Gene symbol, gene name, and annotation symbol from flybase.org. The color-coding system is as follows: Nervous system = blue, External sensory perception = pink, Cuticular = brown, Egg/Reproduction = orange, Mitochondrial = green, Growth/Development = purple, Metal ion binding = teal, Enzyme/Enzymatic activity = red, Other = white, Unknown = gray.
(DOCX)
