# Supplementary material for: Genome-Wide Sequencing and an Open Reading Frame Analysis of Dichlorodiphenyltrichloroethane (DDT) Susceptible (91-C) and Resistant (91-R) Drosophila melanogaster Laboratory Populations
Source: PLoS One. 2014 Jun 10;9(6):e98584. doi: 10.1371/journal.pone.0098584 (PMC4051598; doi:10.1371/journal.pone.0098584)
Supplement: Table S1 — Molecular and biological functions, obtained from uniprot.org and literature searches, for those genes containing SNPs/DIPs from only the 91-R fly line. Gene symbol, gene name, and annotation symbol from flybase.org. The color-coding system is as follows: Nervous system = blue, External sensory perception = pink, Cuticular = brown, Egg/Reproduction = orange, Mitochondrial = green, Growth/Development = purple, Metal ion binding = teal, Enzyme/Enzymatic activity = red, Other = white, Unknown = gray. (DOCX) [file pone.0098584.s004.docx]

| Table S1. Molecular and biological functions, obtained from uniprot.org and literature searches, for those genes containing SNPs/DIPs from only the *91-R* fly line. Gene symbol, gene name, and annotation symbol from flybase.org. The color-coding system is as follows: Nervous system = blue, External sensory perception = pink, Cuticular = brown, Egg/Reproduction = orange, Mitochondrial = green, Growth/Development = purple, Metal ion binding = teal, Enzyme/Enzymatic activity = red, Other = white, Unknown = gray. | | | | | | | |
| --- | --- | --- | --- | --- | --- | --- | --- |
| **Symbol** | **Gene Name** | **Annotation Symbol** | **Chromosome** | **Molecular Function (Gene Ontology) (uniprot.org)** | **Biological Process (uniprot.org)** | **Other Functions (from Journal Articles - See Next Column)** | **Article Citation** |
| CG42329 |  | CG42329 | 2L | Acyltransferase Transferase | --- |  |  |
| CG5556 |  | CG5556 | 2L | Hydrolase Activity | --- |  |  |
| CG9870 |  | CG9870 | 2L | Acyltransferase Transferase | --- |  |  |
| CG3557 |  | CG3557 | 2L | --- | --- |  |  |
| CG18641 |  | CG18641 | 2L | Hydrolase Activity | --- |  |  |
| CG3542 |  | CG3542 | 2L | --- | Nuclear mRNA splicing, via spliceosome |  |  |
| gkt | glaikit | CG8825 | 2L | Exonuclease, Hydrolase, Nuclease | DNA damage, DNA repair | expressed in embryonic central nervous system | Dunlop *et al.*, 2000 |
| E23 | Early gene at 23 | CG3327 | 2L | ATP binding, ATPase Activity | Regulation of circadian rhythm | critical regulatory roles during metamorphosis | Hock *et al.*, 2000 |
| Msp-300 | Muscle-specific protein 300 | CG42768 | 2L | --- | --- | anchors nuclei to actin | Xie & Fischer, 2008 |
| Wnt10 | Wnt10 | CG4971 | 2L | Developmental protein | Wnt signaling pathway |  |  |
| ninaC | neither inactivation nor afterpotential C | CG5125 | 2L | Kinase, Motor protein, Myosin, Serine/threonine-protein kinase, Transferase | Sensory transduction, Vision |  |  |
| CG13791 |  | CG13791 | 2L |  | Inter-male aggressive behavior |  |  |
| fy | fuzzy | CG13396 | 2L |  | Establishment of imaginal disc-derived wing hair orientation; Establishment or maintance of cell polarity |  |  |
| CG9525 |  | CG9525 | 2L | --- | --- |  |  |
| CG32985 |  | CG32985 | 2L | Catalytic activity | --- |  |  |
| chico | chico | CG5686 | 2L | SH2 domain binding; insulin receptor binding; insulin-like growth factor receptor binding; phosphatidylinositol 3-kinase binding | Cell morphogenesis; determination of adult lifespan; germ-line stem-cell niche homeostasis; growth of germarium-derived egg chamber; insulin receptor signaling pathway; Male germ-line stem cell division; Primary spermatocyte growth; Vitellogenesis |  |  |
| CG42818 |  | CG42818 | 2L | --- | --- |  |  |
| CG5888 |  | CG5888 | 2L | --- | --- | putative secreted or transmembrane protein | Zúñiga *et al.*, 2009 |
| CG31812 |  | CG31812 | 2L | Nucleic acid binding; tRNA-intron endonuclease activity | tRNA splicing, via endonucleolytic cleavage and ligation |  |  |
| CadN2 | Cadherin-N2 | CG42829 | 2L | Calcium ion binding | Integral to membrane; Plasma membrane | ** axon extension involved in axon guidance | Prakash *et al.*, 2005 |
| CG42830 |  | CG42830 | 2L | --- | --- |  |  |
| CG7200 |  | CG7200 | 2L | --- | --- |  |  |
| CG7180 |  | CG7180 | 2L | Protein tyrosine phosphatase activity | --- |  |  |
| CG42750 |  | CG42750 | 2L | Dipeptidase activity; Dipeptidyl-peptidase activity; metalloexopeptidase activity | Proteolysis |  |  |
| CG5790 |  | CG5790 | 2L | ATP binding; Receptor signaling protein serine/threonine kinase activity | Phagocytosis, engulfment; Regulation of cell shape |  |  |
| CG43353 |  | CG43353 | 2L | --- | --- |  |  |
| CG43354 |  | CG43354 | 2L | --- | --- |  |  |
| Mst36Fa | Male-specific transcript 36Fa | CG31801 | 2L | --- | Spermatogenesis |  |  |
| tos | tosca | CG10387 | 2L | DNA binding; double-stranded DNA specific 5'-3' exodeoxyribonuclease activity; Flap endonuclease activity; Metal ion binding; Single-stranded DNA specific 5'-3' exodeoxyribonuclease activity | DNA recombination; mismatch repair |  |  |
| CG31751 |  | CG31751 | 2L | Transferase activity; transferring phosphorus-containing groups | --- |  |  |
| msl-1 | male-specific lethal 1 | CG10385 | 2L | DNA binding; chromatin binding; protein binding | Dosage compensation complex assembly involved in dosage compensation by hyperactivation of x chromosome |  |  |
| Jwa | Jwa ortholog | CG10373 | 2L | Ion channel activity | Response to ethanol | Required for acquiring ethanol tolerance | Li *et al.*, 2008 |
| CG17568 |  | CG17568 | 2L | Nucleic acid binding; Zinc ion binding | --- |  |  |
| TepIV | Thiolester containing protein IV | CG10363 | 2L | Endopeptidase inhibitor activity | --- |  |  |
| CG34051 |  | CG34051 | 2L | --- | --- |  |  |
| CG13965 |  | CG13965 | 2L | --- | --- |  |  |
| CG16772 |  | CG16772 | 2L | --- | --- |  |  |
| Nhe2 | Na+/H+ hydrogen exchanger 2 | CG9256 | 2L | Sodium:hydrogen antiporter activity | Regulation of pH |  |  |
| CG8671 |  | CG8671 | 2L | --- | dsRNA transport |  |  |
| dtr | defective transmitter release | CG31623 | 2L | Dynein binding | Cilium morphogenesis; Synaptic transmission |  |  |
| Gr39b | Gustatory receptor 39b | CG31620 | 2L | Taste receptor activity | --- |  |  |
| Ef2b | Elongation factor 2b | CG2238 | 2L | GTP binding; GTPase activity; Translation elongation factor activity | Mitotic spindle elongation |  |  |
| CG7845 |  | CG7845 | 2R | --- | --- |  |  |
| Ars2 |  | CG7843 | 2R | Protein binding | Conversion of ds siRNA to ss siRNA involved in RNA interference; Negative regulation of viral genome replication; nuclear mRNA splicing, via spliceosome; Primary miRNA processing |  |  |
| Tsp42Ee | Tetraspanin 42Ee | CG10106 | 2R | --- | --- |  |  |
| Or43a | Odorant receptor 43a | CG1854 | 2R | --- | --- | odorant binding; olfactory receptor activity | Stortkuhl & Kettler, 2001 |
| Dscam | Down syndrome cell adhesion molecule | CG17800 | 2R | Axon guidance receptor activity; Bacterial cell surface binding; Protein homodimerization activity | Axon extension involved in axon guidance; Axonal fasciculation; Central nervous system morphogenesis; Dendrite self-avoidance; Mushroom body development; Peripheral nervous system development; Phagocytosis |  |  |
| sPLA2 | secretory Phospholipase A2 | CG11124 | 2R | Calcium ion binding; Calcium-dependent phospholipase A2 activity | Lipid catabolic process; Phospholipid metabolic process |  |  |
| Vps13 | Vacuolar protein sorting 13 | CG2093 | 2R | --- | Protein localization |  |  |
| CG1946 |  | CG1946 | 2R | Transferase activity; Transferring acyl groups other than amino-acyl groups | --- |  |  |
| CG30373 |  | CG30373 | 2R | --- | --- |  |  |
| Rs1 | Rs1 | CG2173 | 2R | ATP binding; ATP-dependent helicase activity; Nucleic acid binding; Nucleotidyltransferase | Ribosome biogenesis |  |  |
| CG1773 |  | CG1773 | 2R | Serine-type endopeptidase activity | Proteolysis |  |  |
| CG10459 |  | CG10459 | 2R | --- | --- |  |  |
| CG1698 |  | CG1698 | 2R | Neurotransmitter:sodium symporter activity | --- |  |  |
| CG1688 |  | CG1688 | 2R | Potassium channel activity | --- |  |  |
| dila | dilatory | CG1625 | 2R | --- | --- | sensory neuron cilia | Ma & Jarman, 2011 |
| Cpr47Eb | Cuticular protein 47Eb | CG13224 | 2R | Structural constituent of cuticle | --- |  |  |
| sprt | sprite | CG30023 | 2R | --- | --- |  |  |
| CG8520 |  | CG8520 | 2R | ATP binding; ATPase activity | --- |  |  |
| CG30486 |  | CG30486 | 2R | --- | --- |  |  |
| CG17574 |  | CG17574 | 2R | --- | --- |  |  |
| CG4712 |  | CG4712 | 2R | --- | --- |  |  |
| CG4716 |  | CG4716 | 2R | Methylenetetrahydrofolate dehydrogenase (NAD+) activity | --- |  |  |
| CG17050 |  | CG17050 | 2R | --- | --- |  |  |
| Tfb1 | Tfb1 | CG8151 | 2R |  | DNA repair; Regulation of transcription, DNA-dependent; Transcription, DNA-dependent |  |  |
| CG34184 |  | CG34184 | 2R | --- | --- |  |  |
| Obp50c | Odorant-binding protein 50c | CG30072 | 2R | --- | --- | Odorant binding | Zhou *et al.*, 2004 |
| Obp50b | Odorant-binding protein 50b | CG30073 | 2R | --- | --- | Odorant binding | Zhou *et al.*, 2004 |
| Obp50e | Odorant-binding protein 50e | CG13939 | 2R | --- | --- | Odorant binding | Zhou *et al.*, 2004 |
| Sfp51E | Seminal fluid protein 51E | CG42476 | 2R | --- | --- | Potential seminal fluid gene | Findlay *et al.*, 2008 |
| CG11807 |  | CG11807 | 2R | Phosphatidylinositol binding | Cell communication |  |  |
| CG42391 |  | CG42391 | 2R | --- | --- |  |  |
| CG12963 |  | CG12963 | 2R | --- | --- |  |  |
| clu | clueless | CG8443 | 2R | Binding | Mitochondrion localization |  |  |
| krimp | krimper | CG15707 | 2R | Nucleic acid binding; Zinc ion binding | Karyosome formation; Negative regulation of oskar mRNA translation; Oocyte dorsal/ventral axis specification |  |  |
| CG15708 |  | CG15708 | 2R | DNA binding | --- |  |  |
| Menl-1 | Malic enzyme like-1 | CG7964 | 2R | NAD binding; Malate dehydrogenase (oxaloacetate-decarboxylating) activity; Metal ion binding | Malate metabolic process |  |  |
| Menl-2 | Malic enzyme like-2 | CG7969 | 2R | NAD binding; Malate dehydrogenase (oxaloacetate-decarboxylating) activity; Metal ion binding | Malate metabolic process |  |  |
| CG33960 |  | CG33960 | 2R | --- | --- |  |  |
| CG34190 |  | CG34190 | 2R | --- | --- |  |  |
| mute | muscle wasted | CG34415 | 2R | --- | Muscle cell homeostasis; Regulation of transcription, DNA dependent |  |  |
| CG30461 |  | CG30461 | 2R | --- | --- |  |  |
| ste24c | ste24c prenyl protease type I | CG9002 | 2R | Metalloendopeptidase activity | Proteolysis |  |  |
| ste24b | ste24b prenyl protease type I | CG9001 | 2R | Metalloendopeptidase activity | Proteolysis |  |  |
| CG6796 |  | CG6796 | 2R | ATP binding; Asparagine-tRNA ligase activity; Aspartate-tRNA ligase activity; Nucleic acid binding | Asparaginyl-tRNA aminoacylation |  |  |
| NiPp1 | Nuclear inhibitor of Protein phosphatase 1 | CG8980 | 2R | mRNA binding; Protein binding; Protein serine/threonine phosphatase inhibitor activity | Axon guidance; Negative regulation of protein desphosphorylation |  |  |
| CG6805 |  | CG6805 | 2R | Inositol trisphosphate phosphatase activity | --- |  |  |
| CG15609 |  | CG15609 | 2R | --- | --- |  |  |
| Ark | Apaf-1-related-killer | CG6829 | 2R | ATP binding; Caspase activator activity; Metal ion binding | Activation of caspase activity by cytochrome c; Central nervous system formation; Dendrite morphogenesis; Induction of apoptosis; Positive regulation of compound eye retinal cell programmed cell death; Response to DNA damage stimulus; Salivary gland cell autophagic cell death; Sensory organ development; Sperm individualization |  |  |
| CG9646 |  | CG9646 | 2R | --- | --- |  |  |
| HPS4 | Hermansky-Pudlak Syndrome 4 ortholog | CG4966 | 2R | Thymidylate synthase (FAD) activity | miRNA loading onto RISC involved in gene silencing by miRNA; Negative regulation of gene silencing by RNA; siRNA loading onto RISC involved in chromatin silencing by small RNA |  |  |
| CG5757 |  | CG5757 | 2R | ATP binding; Thymidylate kinase activity | dTDP biosynthetic process |  |  |
| CG10924 |  | CG10924 | 2R | GTP binding; Phosphoenolpyruvate carboxykinase (GTP) activity | Gluconeogenesis |  |  |
| Atg7 | Autophagy-specific gene 7 | CG5489 | 2R | Binding; Catalytic activity | Determination of adult lifespan; Larval midgut cell programmed cell death; Macroautophagy; Regulation of autophagy; Regulation of defense response to virus | requiered for stress resistance, longevity, and neuronal homeostasis | Juhasz & Neufeld, 2008 |
| Gint3 | GDI interacting protein 3 | CG5469 | 2R | --- | --- |  |  |
| CG42306 |  | CG42306 | 2R | --- | --- |  |  |
| CG43070 |  | CG43070 | 2R | --- | --- |  |  |
| CG33454 |  | CG33454 | 2R | --- | --- |  |  |
| CG10081 |  | CG10081 | 2R | Peptidase activity | Proteolysis |  |  |
| CG42753 |  | CG42753 | 2R | --- | --- |  |  |
| Obp56d | Odorant-binding protein 56d | CG11218 | 2R | Odorant binding | Olfactory behavior; Response to pheromone; Sensory perception of smell; Transport |  |  |
| Obp56e | Odorant-binding protein 56e | CG8462 | 2R | Odorant binding | --- |  |  |
| CG16742 |  | CG16742 | 2R | --- | --- |  |  |
| CG15651 |  | CG15651 | 2R | --- | --- |  |  |
| dgt3 | dim ϒ-tubulin 3 | CG3221 | 2R | --- | Mitotic spindle organization; Regulation of mitosis |  |  |
| CG9346 |  | CG9346 | 2R | RNA binding; Nucleotide binding | RNA processing |  |  |
| CG3296 |  | CG3295 | 2R | --- | --- |  |  |
| CG15653 |  | CG15653 | 2R | --- | --- |  |  |
| Cht8 | Cht8 | CG9357 | 2R | Cation binding; Chitin binding; Chitinase activity | Chitin catabolic process |  |  |
| Cht12 | Cht12 | CG30293 | 2R | Cation binding; Chitin binding; Chitinase activity | Chitin catabolic process |  |  |
| Treh | Trehalase | CG9364 | 2R | Alpha,alpha-trehalase activity | Trehalose metabolic process |  |  |
| CG4050 |  | CG4050 | 2R | Binding | --- |  |  |
| CG9406 |  | CG9406 | 2R | Calcium ion binding | --- |  |  |
| Xbp1 | X box binding protein-1 | CG9415 | 2R | Protein dimerization activity; Sequence-specific DNA binding; Sequence-specific DNA binding transcription factor activity | Bristle development; Endoplasmic reticulum unfolded protein response; Wing disc development |  |  |
| CG9418 |  | CG9418 | 2R | DNA binding | --- |  |  |
| CG6758 |  | CG6758 | 2R | --- | --- |  |  |
| CG11170 |  | CG11170 | 2R | ATPase activity | --- |  |  |
| Liprin-γ | Liprin-γ | CG11206 | 2R | Protein homodimerization activity | Axon target recognition; Negative regulation of synaptic growth at neuromuscular junction |  |  |
| ppk12 | pickpocket 12 | CG10972 | 2R | Sodium channel activity | --- |  |  |
| pallidin | pallidin | CG14133 | 3L | Protein binding | --- | ** Eye pigmentation | Cheli *et al.*, 2010 |
| f-cup | flyers-cup | CG9611 | 3R | --- | --- |  |  |
| CG14850 |  | CG14850 | 3R | --- | --- |  |  |
| Cpr100A | Cuticular protein 100A | CG12045 | 3R | Structural constituent of cuticle | --- |  |  |
| CG15545 |  | CG15545 | 3R | --- | --- |  |  |
| onecut | onecut | CG1922 | 4 | Sequence-specific DNA binding; Sequence-specific DNA binding transcription factor activity | --- | nervous system; "may function as a neural-specific transcription factor to regulat certain aspects of neural differentation and possibly to play a role in the maintenance of the neuronal cell phenotype and function." | Nguyen *et al.*, 2000 |
| cals | calsyntenin-1 | CG11059 | 4 | Calcium ion binding | Homophilic cell adhesion | Found in primary & secondary neurons | Fung *et al.*, 2008 |
| ase | asense | CG3258 | X | DNA binding | Bristle morphogenesis; Cell differentiation; Central nervous system development; Peripheral nervous system development; Regulation of transcription, DNA-dependent |  |  |
| CG32816 |  | CG32816 | X | --- | --- |  |  |
| Cyp4g1 | Cytochrome P450-4g1 | CG3972 | X | Electron carrier activity; Heme binding; Monooxygenase activity; Oxidoreductase activity, acting on paired donors, with incorporation or reduction of molecular oxygen | Lipid metabolic process | cuticular | Strychartz *et al.*, Submitted. |
| CG33173 |  | CG33173 | X | ATP binding; ATPase activity; Chitin binding | Chitin metabolic process |  |  |
| dpr18 | dpr18 | CG14948 | X | --- | --- | gustatory | Nakamura *et al.*, 2002 |
| CG9902 |  | CG9902 | X | --- | --- |  |  |
| par-6 | par-6 | CG5884 | X | Protein binding | Apical protein localization; Asymmetric neuroblast division; Border follicle cell migration; Cell adhesion; Establishment of neuroblast polarity; Establishment or maintenance of epithelial cell apical/basal polarity; Estalbishment or maintenance of polarity of embryonic epithelium; Morphogenesis of a polarized epithelium; Negative regulation of protein kinase activity; Oocyte axis specification; Phagocytosis, engulfment; Regulation of cell shape; Synapse assembly; Zonula adherens assembly |  |  |
| CG34328 |  | CG34328 | X | --- | --- |  |  |
| CG32549 |  | CG32549 | X | 5'-nucleotidase activity; Metal ion binding | --- |  |  |
| CG32507 |  | CG32507 | X | --- | --- |  |  |
| shakB | shaking B | CG34358 | X | Gap junction channel activity; Ion channel activity; Photoreceptor activity | Gap junction assembly; Jump response; Phototransduction |  |  |
