# Supplementary material for: Genome-Wide Sequencing and an Open Reading Frame Analysis of Dichlorodiphenyltrichloroethane (DDT) Susceptible (91-C) and Resistant (91-R) Drosophila melanogaster Laboratory Populations
Source: PLoS One. 2014 Jun 10;9(6):e98584. doi: 10.1371/journal.pone.0098584 (PMC4051598; doi:10.1371/journal.pone.0098584)
Supplement: Table S2 — Molecular and biological functions, obtained from uniprot.org and literature searches, for those genes containing SNPs/DIPs from only the 91-C fly line. Gene symbol, gene name, and annotation symbol from flybase.org. The color-coding system is as follows: Nervous system = blue, External sensory perception = pink, Cuticular = brown, Egg/Reproduction = orange, Mitochondrial = green, Growth/Development = purple, Metal ion binding = teal, Enzyme/Enzymatic activity = red, Other = white, Unknown = gray. (DOCX) [file pone.0098584.s005.docx]

| Table S2. Molecular and biological functions, obtained from uniprot.org and literature searches, for those genes containing SNPs/DIPs from only the *91-C* fly line. Gene symbol, gene name, and annotation symbol from flybase.org. The color-coding system is as follows: Nervous system = blue, External sensory perception = pink, Cuticular = brown, Egg/Reproduction = orange, Mitochondrial = green, Growth/Development = purple, Metal ion binding = teal, Enzyme/Enzymatic activity = red, Other = white, Unknown = gray. | | | | | | | |
| --- | --- | --- | --- | --- | --- | --- | --- |
| **Symbol** | **Gene Name** | **Annotation Symbol** | **Chromosome** | **Molecular Function (Gene Ontology) (uniprot.org)** | **Biological Process (uniprot.org)** | **Other Functions (from Journal Articles - See Next Column)** | **Article Citation** |
| I(1)1Bi | lethal (1) 1Bi | CG6189 | X | DNA binding; DNA-directed DNA polymerase activity | transcription, DNA-dependent |  |  |
| CG11403 |  | CG11403 | X | ATP binding; ATP-dependent DNA helicase activity; DNA binding |  |  |  |
| Ocri | Oculocerebrorenal syndrome of Lowe 1 ortholog | CG3573 | X | inositol, 1,3,4,5-tetrakisphosphate 5 phosphatase activity; inositol-1,4,5-trisphosphate 5-phosphatase activity; inositol-polyphosphate 5-phosphatase activity | contractile ring localization involved in cell cycle cytokinesis; endocytosis; regulation of cytokinesis, actomyosin contractile ring assembly; signal transduction |  |  |
| mit(1)15 | mitotic 15 | CG9900 | X |  | cell division; meiotic anaphase I; mitotic cell cycle spindle assembly checkpoint; mitotic sister chromatid segregation; regulation of meiosis |  |  |
| CG2861 |  | CG2861 | X |  |  |  |  |
| CG4593 |  | CG4593 | X |  |  |  |  |
| CG32702 |  | CG32702 | X | calcium ion binding |  |  |  |
| Cht6 | Cht6 | CG43374 | X | cation binding; chitin binding; chitinase activity | chitin catabolic process |  |  |
| Myo10A | unconventional myosin class XV | CG2174 | X | ATP binding; motor activity; protein binding | dorsal closure; filopodium assembly; intracellular protein transport |  |  |
| CG16922 |  | CG16922 | X |  |  | protein binding; ATPase activity, coupled; ATP binding; motor activity | Liu *et al.*, 2008 |
| hop | hopscotch | CG1594 | X | ATP binding; non-membrane spanning protein tyrosine kinase activity | STAT protein import into nucleus; apical constriction; border follicle cell migration; cellular defense response; compound eye photoreceptor cell differentiation; cytokinesis; defense response to virus; encapsulation of foreign target; equator specification; eye-antennal disc morphogenesis; germ-line stem cell division; hemocyte proliferation; hindgut morphogenesis; humoral immune response; imaginal disc-derived leg morphogenesis; imaginal disc-derived wing morphogenesis; llamellocyte differentiation; long-term memory; mediolaterial intercalcation; ommatidial rotation; oepn tracheal system development; ovarian follicle cell stalk formation; periodic partitioning; primary sex determination; regulation of JAK-STAT cascade; regulation of embryonic cell shape; regulation of hemocyte differentiation; regulation of transcription, DNA-dependent; somatic stem cell division; stem cell maintenance; transcription, DNA-dependent; tyrosine phosphorylation of STAT protein |  |  |
| CG17209 |  | CG17209 | X | DNA binding; DNA-directed RNA polymerase activity; ribonucleoside binding; zinc ion binding |  |  |  |
| f | forked | CG42864 | X |  |  | bristle morphology; sensory perception of sound | Cosetti *et al.*, 2008 |
| CG12991 |  | CG12991 | X |  |  |  |  |
| CG12992 |  | CG12992 | X |  |  |  |  |
| CG14204 |  | CG14204 | X | transferase activity, transferring acyl groups other than amino-acyl groups | chaeta development; wing disc development |  |  |
| CG1801 |  | CG1801 | X | ATP binding; ATPase activity |  |  |  |
| Pros45 | Pros45 | CG1489 | X | ATP binding; endopeptidase activity; nucleoside-triphosphatase activity | Protein catabolic process; proteolysis; response to DNA damage stimulus |  |  |
| CG15580 |  | CG15580 | 3R |  |  |  |  |
| α-Est6 | α-Esterase-6 | CG1108 | 3R | Carboxylesterase activity; methyl indole-3-acetate esterase activity; methyl jasmonate esterase activity; methyl salicylate esterase activity |  |  |  |
| α-Est4aψ | α-Est4aψ | CR31511 | 3R |  |  |  |  |
| CG42375 |  | CG42375 | 3R |  |  |  |  |
| CG9288 |  | CG9288 | 3R |  | glycine catabolic process |  |  |
| CG9286 |  | CG9286 | 3R |  |  |  |  |
| Dip-B | Dipeptidase B | CG9285 | 3R | aminopeptidase activity; dipeptidyl-peptidase activity; manganese ion binding; metalloexopeptidase activity; tripeptidyl-peptidase activity | proteolysis |  |  |
| CG15695 |  | CG15695 | 3R | catalytic activity |  |  |  |
| grass | Gram-positive Specific Serine Protease | CG5896 | 3R | serine-type endopeptidase activity | defense response to Gram-positive bacterium; innate immune response; positive regulation fo Toll signaling pathway; proteolysis |  |  |
| CG42558 |  | CG42558 | 3R |  |  |  |  |
| CG42557 |  | CG42557 | 3R |  |  |  |  |
| CG15537 |  | CG15537 | 3R |  |  |  |  |
| cindr | CIN85 and CD2AP orthologue | CG31012 | 3R | actin filament organization; border follicle cell migration; compound eye morphogenesis; cytokinesis; intercellular bridge organization; positive regulation of receptor-mediated endocytosis | SH3 domain binding |  |  |
| 5-HT7 | Serotonin receptor 7 | CG12073 | 3R | activation of adenylate cyclase activity by serotonin receptor signaling pathway; female mating behavior; male courtship behavior, orientation prior to leg tapping and wing vibration; male courtship behavior, proboscis-mediated licking; male courtship behavior, veined wing vibration | dopamine receptor activity; serotonin receptor activity |  |  |
| CG31008 |  | CG31008 | 3R |  |  |  |  |
| CG11317 |  | CG11317 | 3R | zinc ion binding |  |  |  |
| CG11318 |  | CG11318 | 3R | G-protein coupled receptor activity | neuropeptide signaling pathway |  |  |
| Gycβ100B | Guanylyl cyclase β-subunit at 100B | CG1470 | 3R | guanylate cyclase activity; heme binding; nucleotide binding | intracellular signal transduction |  |  |
| CG31004 |  | CG31004 | 3R |  | cell-matrix adhesion |  |  |
| chp | chaoptic | CG1744 | 3R |  | homophilic cell adhesion; response to stimulus; rhabdomere development; visual perception |  |  |
| CG18673 |  | CG18673 | 3R | carbonate dehydratase activity; zinc ion binding | one-carbon metabolic process |  |  |
| CG15555 |  | CG15555 | 3R | sodium channel activity |  |  |  |
| CycG | Cyclin G | CG11525 | 3R | transferase activity | negative regulation of G1/S transition of mitotic cell cycle; negative regulation of S phase of mitotic cell cycle |  |  |
| CG5964 |  | CG5964 | 3L |  |  |  |  |
| Klp68D | Kinesin-like protein at 68D | CG7293 | 3L | ATP binding; microtubule motor activity | anterograde axon cargo transport; establishment or maintenance of microtubule cytoskeleton polarity; sensory perception of smell |  |  |
| Grip163 | Grip163 | CG5688 | 3L | mitosis; mitotic spindle organization | gamma-tubulin binding |  |  |
| CG32100 |  | CG32100 | 3L |  |  |  |  |
| CG14590 |  | CG14590 | 2R | zinc ion binding | negative regulation of transcription, DNA-dependent |  |  |
| Pld | Phospholipase D | CG12110 | 2R | Phosphatidylinositol binding; phospholipase D activity | Golgi organization; cellularization; gastrulation involving germ band extension; phototransduction; positive regulation of Golgi vesicle fusion to target membrane |  |  |
| l(2)01289 | lethal (2) 01289 | CG9432 | 2R | protein disulfide isomerase activity | cell redox homeostasis |  |  |
| wech | wech | CG42396 | 2R | zinc ion binding | cell differentiation; instar larval development; muscle attachment |  |  |
| Gr43b | Gr43b | CG1339 | 2R | G-protein coupled receptor activity | sensory perception of chemical stimulus |  |  |
| MLh1 | Mlh1 | CG11482 | 2R | ATP binding; ATPase activity; mismatched DNA binding | mismatch repair; reciprocal meiotic recombination |  |  |
| Rad51C | Rad51C | CG2412 | 2R | ATP binding; DNA binding; DNA-dependent ATPase activity | DNA metabolic process |  |  |
| CG42382 |  | CG42382 | 2R | nucleic acid binding; zinc ion binding |  |  |  |
| l(2)k10201 | lethal (2) k10201 | CG13951 | 2R | zinc ion binding | multicellular organismal development |  |  |
| wun | wunen | CG8804 | 2R | phosphatidate phosphatase activity; protein homodimerization activity | embryonic pattern specification; germ cell programmed cell death; germ cell repulsion; pole cell migration |  |  |
| CG12129 |  | CG12129 | 2R | RNA binding; catalytic activity | RNA metabolic process; regulation of transcription, DNA-dependent |  |  |
| sel | seele | CG12918 | 2R |  |  | polarity specification of dorsal/ventral axis; regulation of protein processing; regulation of protein secretion | Stein *et al.*, 2010 |
| mms4 |  | CG12936 | 2R | DNA binding; nuclease activity | DNA metabolic process |  |  |
| CG12942 |  | CG12942 | 2R | nucleic acid binding; zinc ion binding |  |  |  |
| CG8290 |  | CG8290 | 2R |  |  |  |  |
| Rnrs | Ribonucleoside diphosphate reductase small subunit | CG8975 | 2R | DNA replication; activation of caspase activity; deoxyribonucleoside diphosphate metabolic process | ribonucleoside-diphosphate reductase activity |  |  |
| rho-7 | rhomboid-7 | CG8972 | 2R | serine-type endopeptidase activity | mitochondrial fusion; proteolysis |  |  |
| CG8298 |  | CG8298 | 2R | glycerol kinase activity | glycerol-3-phosphate metabolic process |  |  |
| CG30046 |  | CG30046 | 2R |  |  |  |  |
| CG30047 |  | CG30047 | 2R | peptidase activity | proteolysis |  |  |
| Cpr49Ah | Cuticular protein 49Ah | CG8515 | 2R | structural constituent of cuticle |  |  |  |
| CG8525 |  | CG8525 | 2R | deoxyribose-phosphate aldolase activity | deoxyribonucleotide catabolic process |  |  |
| DH44-R2 | Diuretic hormone 44 receptor 2 | CG12370 | 2R | G-protein coupled receptor activity; diuretic hormone receptor activity |  |  |  |
| CG34234 |  | CG34234 | 2R |  |  |  |  |
| CG8646 |  | CG8646 | 2R | N-acetylgalactosamine-4-sulfatase activity |  |  |  |
| CG13148 |  | CG13148 | 2R |  |  |  |  |
| Aats-val | Valyl-tRNA synthetase | CG4062 | 2R | ATP binding; glutamate-tRNA ligase activity; valine-tRNA ligase activity | valyl-tRNA aminoacylation |  |  |
| CG34315 |  | CG34315 | 2R |  |  |  |  |
| lat | latheo | CG4088 | 2R | DNA binding | DNA-dependent DNA replication initiation; larval feeding behavior; olfactory learning |  |  |
| CG4734 |  | CG4734 | 2R |  |  |  |  |
| CG6145 |  | CG6145 | 2R | NAD+ kinase activity |  |  |  |
| CG34442 |  | CG34442 | 2R |  |  |  |  |
| CG17385 |  | CG17385 | 2R | nucleic acid binding; zinc ion binding |  |  |  |
| CG12863 |  | CG12863 | 2R | nucleic acid binding; zinc ion binding |  |  |  |
| CG12853 |  | CG12853 | 2R |  |  |  |  |
| CG10253 |  | CG10253 | 2R | UDP-N-acetylmuramate dehydrogenase activity; alkylglycerone-phosphate synthase activity; flavin adenine dinucleotide binding | lipid biosynthetic process |  |  |
| CG8089 |  | CG8089 | 2R | nucleic acid binding; zinc ion binding |  |  |  |
| igl | igloo | CG18285 | 2R | calmodulin binding |  |  |  |
| CG43068 |  | CG43068 | 2R |  |  |  |  |
| CG8180 |  | CG8180 | 2R |  |  |  |  |
| CG12964 |  | CG12964 | 2R |  |  |  |  |
| Ir52d | ionotropic receptor 52d | CG30464 | 2R | receptor activity |  |  |  |
| Khc-73 | Kinesin-73 | CG8183 | 2R | ATP binding; microtubule motor activity; protein homodimerization activity | establishment of spindle orientation; microtubule-based movement; regulation of synapse structure and activity |  |  |
| CG30467 |  | CG30467 | 2R | binding | acute-phase response |  |  |
| CG8187 |  | CG8187 | 2R |  |  |  |  |
| CG8192 |  | CG8192 | 2R | chitin binding | chitin metabolic process |  |  |
| CG30466 |  | CG30466 | 2R |  |  |  |  |
| Cng | Cyclic-nucleotide-gated ion channel protein | CG42701 | 2R | cGMP binding; ion channel activity | cGMP-mediated signaling |  |  |
| Nox | NADPH oxidase | CG34399 | 2R | calcium ion binding; electron carrier activity; flavin adenine dinucleotide binding; iron ion binding; oxidoreductase activity |  |  |  |
| CG8060 |  | CG8060 | 2R |  |  |  |  |
| CG4409 |  | CG4409 | 2R |  |  |  |  |
| CG8963 |  | CG8963 | 2R | binding | RNA metabolic process; brain morphogenesis; inter-male aggressive behavior; locomotion involved in locomotory behavior; olfactory behavior; startle response |  |  |
| CG5009 |  | CG5009 | 2R | acyl-CoA dehydrogenase activity; acyl-CoA oxidase activity; flavin adenine dinucleotide binding | fatty acid beta-oxidation; generation of precursor metabolites and energy; prostaglandin metabolic process |  |  |
| CG14491 |  | CG14491 | 2R |  |  |  |  |
| elk | eag-like K+ channel | CG5076 | 2R | two-component sensor activity; voltage-gated potassium channel activity | regulation of transcription, DNA-dependent |  |  |
| CG5098 |  | CG5098 | 2R |  |  |  |  |
| Ote | Otefin | CG5581 | 2R | transcription corepressor activity; transcription factor binding | female germ-line stem cell division; germ-line stem cell maintenance; negative regulation of transcription, DNA-dependent; nuclear envelope reassembly; positive regulation of BMP signaling pathway |  |  |
| CG18536 |  | CG18536 | 2R |  |  |  |  |
| sbb | scribbler | CG5580 | 2R | transcription corepressor activity | axon guidance; axon target recognition; imaginal disc-derived wing morphogenesis; larval locomotory behavior; negative regulation of smoothened signaling pathway; negative regulation of transcription from RNA polymerase II promoter; regulation of imaginal disc growth; wing disc dorsal/ventral pattern formation |  |  |
| CG15087 |  | CG15087 | 2R |  |  |  |  |
| Dpt | Diptericin | CG12763 | 2R |  | antibacterial humoral response; defense response to Gram-negative bacterium; innate immune response |  |  |
| endoB | endophilin B | CG9834 | 2R |  |  | associate with membranes of the early secretory pathway; bind to acidic phospholipids and tubulate liposomes; exhibit LPA-AT | Huttner & Schmidt, 2002 |
| CG13872 |  | CG13872 | 2R |  |  |  |  |
| RpS18 | Ribosomal protein S18 | CG8900 | 2R | rRNA binding; structural constituent of ribosome | mitotic spindle elongation; ribosome biogenesis; translation |  |  |
| CG8908 |  | CG8908 | 2R | ATP binding; ATPase activity |  |  |  |
| CG30148 |  | CG30148 | 2R |  |  |  |  |
| hbn | homeobrain | CG33152 | 2R | sequence-specific DNA binding; sequence-specific DNA binding transcription factor activity |  |  |  |
| CG15650 |  | CG15650 | 2R |  |  |  |  |
| CG9313 |  | CG9313 | 2R | ATPase activity |  |  |  |
| dom | domino | CG9696 | 2R | ATP binding; DNA binding; helicase activity; protein binding | cell cycle; dendrite guidance; gene silencing; hemopoiesis; histone acetylation; histone exchange; instar larval or pupal development; negative regulation of hemocyte proliferation; oogenesis; positive regulation of Notch signaling pathway; regulation of alternative nuclear mRNA splicing, via spliceosome; regulation of transcription, DNA-dependent; transcription, DNA-dependent; wing disc pattern formation |  |  |
| Egfr | Epidermal growth factor receptor | CG10079 | 2R | ATP binding; epidermal growth factor-activated receptor activity; protein binding | G2/M transition of mitotic cell cycle; Malpighian tubule morphogenesis; R7 cell differentiation; antennal development; anti-apoptosis; behavioral response to ethanol; border follicle cell migration; brain development; branched duct epithelial cell fate determination, open tracheal system; cell projection assembly; cell-cell adhesion; chorion-containing eggshell pattern formation; compound eye cone cell fate commitment; compound eye photoreceptor fate commitment; determination of genital disc primordium; dorsal appendage formation; dorsal closure; establishment or maintenance of apical/basal cell polarity; eye-antennal disc morphogenesis; female germ-line cyst encapsulation; gastrulation; germ-band shortening; germ-line stem cell maintenance; gonad development; haltere development; heart process; imaginal disc-derived wing vein morphogenesis; imaginal disc-derived wing vein specification; leg disc proximal/distal pattern formation; maintenance of epithelial integrity, open tracheal system; male germ-line cyst encapsulation; maternal determination of dorsal/ventral axis, ovarian follicular epithelium, soma encoded; muscle attachment; muscle cell fate specification; negative regulation of S phase of mitotic cell cycle; negative regulation of compound eye retinal cell programmed cell death; notum cell fate specification; notum development; oenocyte differentiation; ommatidial rotation; oocyte anterior/posterior axis specification; oocyte dorsal/ventral axis specification; peripheral nervous system development; positive regulation of cell proliferation; progression of morphogenetic furrow involved in compound eye morphogenesis; regulation of R8 cell spacing in compound eye; regulation of hemocyte differentiation; salivary gland development; second mitotic wave involved in compound eye morphogenesis; segment polarity determination; spiracle morphogenesis, open tracheal system; stomatogastric nervous system development; tracheal pit formation in open tracheal system; wing disc proximal/distal pattern formation |  |  |
| clt | cricklet | CG9858 | 2R | carboxylesterase activity; methyl indole-3-acetate esterase activity; methyl jasmonate esterase activity; methyl salicylate esterase activity | male mating behavior |  |  |
| rad50 | rad50 | CG6339 | 2R | ATP binding; double-stranding DNA binding; nuclease activity; zinc ion binding | doulbe-strand break repair; meiosis; protein localization; telomere capping; telomere maintenance via recombination |  |  |
| CG30195 |  | CG30195 | 2R |  |  |  |  |
| CG9826 |  | CG9826 | 2R |  | transmembrane transport |  |  |
| CG11555 |  | CG11555 | 2L |  |  |  |  |
| smo | smoothened | CG11561 | 2L | G-protein coupled receptor activity; PDZ domain binding; Wnt-activated receptor activity; Wnt-protein binding; hedgehog receptor activity | Bolwig's organ morphogenesis; anterior/posterior lineage restriction, imaginal disc; axon extension involved in axon guidance; blastoderm segmentation; canonical Wnt receptor signaling pathway; cardioblast differentiation; cerebellar cortex morphogenesis; ciliary receptor clustering involved in smoothened signaling pathway; determination of left/right symmetry; eye-antennal disc morphogenesis; gonad development; heart morphogenesis; negative regulation of S phase of mitotic cell cycle; negative regulation of apoptosis; neuron fate commitment; neuron projection regeneration; ovarian follicle cell development; positive regulation of neuroblast proliferation; positive regulation of smoothened signaling pathway; smoothened signaling pathway involved in regulation of cerebellar granule cell precursor cell proliferation; somatic stem cell maintenance; vasculogenesis; wing disc anterior/posterior pattern formation |  |  |
| a5 | antennal protein 5 | CG5430 | 2L |  |  | Odorant binding protein; antennal protein | Swarup *et al.*, 2011 |
| CG31924 |  | CG31924 | 2L | hydrolase activity |  |  |  |
| CG14352 |  | CG14352 | 2L | DNA binding |  |  |  |
| RFeSP | Rieske iron-sulfur protein | CG7361 | 2L | 2 iron, 2 sulfer cluster binding; ubiquinol-cytochrome-c reductase activity |  |  |  |
| CG31937 |  | CG31937 | 2L | binding; oxidoreductase activity |  |  |  |
| Nplp4 | Neuropeptide-like precursor 4 | CG15361 | 2L | neuropeptide hormone activity | neuropeptide signaling pathway |  |  |
| CG4238 |  | CG4238 | 2L | ubiquitin-protein ligase activity | protein ubiquitination involved in ubiquitin-dependent protein catabolic process |  |  |
| Uch | Ubiquitin carboxy-terminal hydrolase | CG4265 | 2L | cysteine-type peptidase activity; ubiquitin thiolesterase activity | protein deubiquitination; ubiquitin-dependent protein catabolic process |  |  |
| CG34174 |  | CG34174 | 2L |  |  |  |  |
| CG10880 |  | CG10880 | 2L | binding |  |  |  |
| CG7082 |  | CG7082 | 2L | RNA binding |  |  |  |
| CG4267 |  | CG4267 | 2L | hydrolase activity | lipid metabolic process |  |  |
| CG31686 |  | CG31686 | 2L |  |  |  |  |
| CG43099 |  | CG43099 | 2L |  |  |  |  |
| CG42658 |  | CG42658 | 2L |  |  |  |  |
| Cyp309a1 | Cyp309a1 | CG9964 | 2L | electron carrier activity; heme binding; monooxygenase activity; oxidoreductase activity, acting on paired donors, with incorporation or reduction of molecular oxygen |  |  |  |
| CG9663 |  | CG9663 | 2L | ATP binding; ATPase activity |  |  |  |
| Ndae1 | Na+ -driven anion exchanger 1 | CG42253 | 2L | inorganic anion exchange activity | bicarbonate transport; chloride transport; proton transport; sodium ion transport |  |  |
| CG42533 |  | CG42533 | 2L | GTP binding; GTPase binding; guanyl-nucleotide exchange factor activity |  |  |  |
| Proct | Proctolin | CG7105 | 2L | neuropeptide hormone activity |  |  |  |
| wol | wollknaeuel | CG7870 | 2L | dolichyl-phosphate beta-glucosyltransferase activity | Golgi organization; embryonic pattern specification; segment specification | chitin-based cuticle development | Shaik *et al.*, 2011 |
| Scgα | Sarcoglycan α | CG7851 | 2L | calcium ion binding | sarcoglycan complex |  |  |
| CG7840 |  | CG7840 | 2L | oxidoreductase activity, acting on CH-CH group of donors | dolichol metabolic process; dolichol-linked oligosaccharide biosynthetic process; polyprenol catabolic process |  |  |
| CG7810 |  | CG7810 | 2L |  |  |  |  |
| CG7806 |  | CG7806 | 2L | ATP binding; xenobiotic-transporting ATPase activity |  |  |  |
| CG31898 |  | CG31898 | 2L |  |  |  |  |
| Tsp29Fa | Tetraspanin 29Fa | CG9494 | 2L |  |  |  |  |
| CG12439 |  | CG12439 | 2L |  |  |  |  |
| c(2)M | crossover suppressor on 2 of Manheim | CG4249 | 2L |  | resolution of meiotic recombination intermediates |  |  |
| beat-IIIa | beat-IIIa | CG12621 | 2L |  |  |  |  |
| CG34169 |  | CG34169 | 2L |  |  |  |  |
| Gr36a | Gustatory receptor 36a | CG31747 | 2L | taste receptor activity |  |  |  |
| Gr36c | Gustatory receptor 36c | CG31748 | 2L | taste receptor activity |  |  |  |
| GR36d | Gr36d | CG31750 | 2L | G-protein coupled receptor activity | sensory perception of chemical stimulus |  |  |
| CG6380 |  | CG6380 | 2L | protein phosphatase inhibitor activity | regulation of phosphoprotein phosphatase activity; regulation of signal transduction |  |  |
| CLIP-190 | Cytoplasmic linker protein 190 | CG5020 | 2L | actin binding; microtubule binding | cellularization |  |  |
| CG15141 |  | CG15141 | 2L | ubiquitin-protein ligase activity; zinc ion binding |  |  |  |
| CG10178 |  | CG10178 | 2L |  |  |  |  |
| amos | absent MD neurons and olfactory sensilla | CG10393 | 2L |  | nervous system development; regulation of transcription, DNA-dependent; sensory organ precursor cell fate determination; transcription, DNA-dependent |  |  |
| CG42848 |  | CG42848 | 2L |  |  |  |  |
| l(2)37Cb | lethal (2) 37Cb | CG10689 | 2L | ATP binding; ATP-dependent helicase activity; nucleic acid binding; nucleotidyltransferase activity | nuclear mRNA splicing, via spliceosome |  |  |
| l(2)37Cd | lethal (2) 37Cd | CG10563 | 2L |  | phagocytosis, engulfment |  |  |
| Aats-asn | Asparaginyl-tRNA synthetase | CG10687 | 2L | asparaginyl-tRNA aminoacylation | ATP binding; asparagine-tRNA ligase activity; aspartate-tRNA ligase activity; nucleic acid binding |  |  |
| CG10462 |  | CG10462 | 2L | nucleic acid binding; zinc ion binding |  |  |  |
| CG10631 |  | CG10631 | 2L | nucleic acid binding; zinc ion binding |  |  |  |
| CG10664 |  | CG10664 | 2L | Golgi organization; cell proliferation; mitotic cell cycle | cytochrome-c oxidase activity |  |  |
| CG42866 |  | CG42866 | 2L |  |  |  |  |
| CG17570 |  | CG17570 | 2L |  |  |  |  |
| CheB38b | Chemosensory protein B 38b | CG33321 | 2L |  | detection of pheromone |  |  |
