# Supplementary material for: Genome-Wide Sequencing and an Open Reading Frame Analysis of Dichlorodiphenyltrichloroethane (DDT) Susceptible (91-C) and Resistant (91-R) Drosophila melanogaster Laboratory Populations
Source: PLoS One. 2014 Jun 10;9(6):e98584. doi: 10.1371/journal.pone.0098584 (PMC4051598; doi:10.1371/journal.pone.0098584)
Supplement: Table S3 — Molecular and biological functions, obtained from uniprot.org and literature searches, for those genes containing SNPs/DIPs from both the 91-C and 91-R fly line. Gene symbol, gene name, and annotation symbol from flybase.org. The color-coding system is as follows: Nervous system = blue, External sensory perception = pink, Cuticular = brown, Egg/Reproduction = orange, Mitochondrial = green, Growth/Development = purple, Metal ion binding = teal, Enzyme/Enzymatic activity = red, Other = white, Unknown = gray. (DOCX) [file pone.0098584.s006.docx]

| Table S3. Molecular and biological functions, obtained from uniprot.org and literature searches, for those genes containing SNPs/DIPs from both the *91-C* and *91-R* fly line. Gene symbol, gene name, and annotation symbol from flybase.org. The color-coding system is as follows: Nervous system = blue, External sensory perception = pink, Cuticular = brown, Egg/Reproduction = orange, Mitochondrial = green, Growth/Development = purple, Metal ion binding = teal, Enzyme/Enzymatic activity = red, Other = white, Unknown = gray. | | | | | | | |
| --- | --- | --- | --- | --- | --- | --- | --- |
| **Symbol** | **Gene Name** | **Annotation Symbol** | **Chromosome** | **Molecular Function (Gene Ontology) (uniprot.org)** | **Biological Process (uniprot.org)** | **Other Functions (from Journal Articles - See Next Column)** | **Article Citation** |
| CG31921 |  | CG31921 | 2L |  |  |  |  |
| Plc21C | Phospholipase C at 21C | CG4574 | 2L | calcium ion binding; phosphatidylinositol phospholipase C activity; signal transducer activity | flight behavior; intracellular signal transduction; lipid catabolic process |  |  |
| CG5440 |  | CG5440 | 2L | ATP binding; ubiquitin-protein ligase activity |  |  |  |
| haf | hattifattener | CG14351 | 2L |  | motor axon guidance |  |  |
| CG31935 |  | CG31935 | 2L | Rab GTPase activator activity; Rab GTPase binding |  |  |  |
| Eno | Enolase | CG17654 | 2L | Magnesium ion binding; Phosphopyruvate hydratase activity | Glycolysis |  |  |
| Rrp40 |  | CG31938 | 2L | Hydrolase activity |  |  |  |
| CG31663 |  | CG31663 | 2L |  |  |  |  |
| CG7295 |  | CG7295 | 2L | Heme binding |  |  |  |
| CG15385 |  | CG15385 | 2L | Acid phosphatase activity |  |  |  |
| mio | missing oocyte | CG7074 | 2L |  | Double-strand break repair; Multicellular organismal development; oocyte differentiation; oocyte fate determination; regulation of meiosis |  |  |
| Vglut | Vesicular glutamate transporter | CG9887 | 2L | L-glutamate transmembrane transporter activity; neurotransmitter transporter activity | synaptic transmission, glutamatergic |  |  |
| Cyp309a2 | Cyp309a2 | CG18559 | 2L | electron carrier activity; heme binding; monooxygenase activity; oxidoreductase activity, acting on paired donors, with incorporation or reduction of molecular oxygen |  |  |  |
| CG17264 |  | CG17264 | 2L |  |  |  |  |
| CG3605 |  | CG3605 | 2L |  | nuclear mRNA splicing, via spliceosome |  |  |
| toc | toucan | CG9660 | 2L | hydrolase activity | ovarian follicle cell development; syncytial blastoderm mitotic cell cycle |  |  |
| CG13786 |  | CG13786 | 2L |  |  |  |  |
| CG5149 |  | CG5149 | 2L | structural molecule activity |  |  |  |
| CG15818 |  | CG15818 | 2L | binding |  |  |  |
| chm | chameau | CG5229 | 2L | histone acetyltransferase activity; sequence-specific DNA binding transcription factor activity; transcription coactivator activity; zinc ion binding | dendrite morphogeneisis; gene silencing; negative regulation of transcription, DNA-dependent; wing disc dorsal/ventral pattern formation |  |  |
| Gr28b | Gustatory receptor 28b | CG13788 | 2L | taste receptor activity | feeding behavior; melanization defense response |  |  |
| Spn7 | Serine protease inhibitor 7 | CG6717 | 2L | peptidase activity; serine-type endopeptidase inhibitor activity | proteolysis; regulation of proteolysis |  |  |
| spz3 | spatzle 3 | CG7104 | 2L |  |  | Toll binding; Toll signaling pathway | Parker *et al*., 2001 |
| Spn28Da | Serpin 28Da | CG31902 | 2L | serine-type endopeptidase inhibitor activity |  |  |  |
| d | dachs | CG42840 | 2L | ATP binding; ATPase activity; motor activity |  | establishment of ommatidial planar polarity; imaginal disc-derived wing vein morphogenesis; leg disc development; wing disc development | Mao *et al.,* 2006 |
| CG34398 |  | CG34398 | 2L |  |  |  |  |
| osp | outspread | CG3479 | 2L |  |  |  |  |
| heix | heixuedian | CG5876 | 2L | prenyltransferase activity | menaquinone biosynthetic process |  |  |
| mRpL4 | mitochondrial ribosomal protein L4 | CG5818 | 2L | structural constituent of ribosome | translation |  |  |
| CG42389 |  | CG42389 | 2L |  |  |  |  |
| CG31815 |  | CG31815 | 2L |  |  |  |  |
| CG31782 |  | CG31782 | 2L | nucleic acid binding; zinc ion binding |  |  |  |
| Ugt36Bb | Ugt36Bb | CG13271 | 2L | glucuronosyltransferace activity |  | polysaccharide metabolic process, defense response, steroid metabolic process, response to toxin | Sambandan *et al.*, 2008 |
| CG13272 |  | CG13272 | 2L |  |  |  |  |
| CG13280 |  | CG13280 | 2L | binding; oxidoreductase activity |  |  |  |
| mdy | midway | CG31991 | 2L | cholesterol O-acyltransferase activity; diacylglycerol O-acyltransferase activity | Negative regulation of lipid storage; oogenesis; regulation of nurse cell apoptosis; wing disc development |  |  |
| tweek | tweek | CG42555 | 2L |  | Synaptic vesicle endocytosis |  |  |
| CG42556 |  | CG42556 | 2L |  |  |  |  |
| CG31784 |  | CG31784 | 2L |  |  |  |  |
| Gr36b | Gustatory receptor 36b | CG31744 | 2L | Taste receptor activity | Integral to membrane; plasma membrane |  |  |
| Lrch | Leucine-rich-repeats and calponin homology domain protein | CG6860 | 2L |  |  | stabilization of the cell cortex during cell division; cytoskeletal scaffolding protein; cleavage furrow | Foussard *et al.*, 2010 |
| Dif | Dorsal-related immunity factor | CG6794 | 2L | sequence-specific DNA binding; sequence-specific DNA binding transcription factor activity | Toll signaling pathway; antifuntal peptide production; defense response to Gram-positive bacterium; innate immune response; lamellocyte differentation; peripheral nervous system neuron development; plasmatocyte differentiation; positive regulation of antifungal peptide biosynthetic process; positive regulation of transcritpion from RNA polymerase II promoter; regulation of hemocyte proliferation; response to DNA damage stimulus; salivary gland histolysis |  |  |
| Sytα | Synaptotagmin α | CG5559 | 2L | Calcium-dependent phospholipid binding | Neurotransmitter secretion; vesicle-mediated transport |  |  |
| CadN | Cadherin-N | CG7100 | 2L | beta-catenin binding; calcium ion binding | R7 cell development; R8 cell development; axon extension involved in axon guidance; axon extension involved in development; axon target recognition; axonal fasciculation; calcium-dependent cell-cell adhesion; homophilic cell adhesion; negative regulation of dendrite morphogenesis; ommatidial rotation; regulation of axon extension involved in axon guidance; retinal ganglion cell axon guidance |  |  |
| CG17681 |  | CG17681 | 2L | N-acetyltransferase activity |  |  |  |
| CG15160 |  | CG15160 | 2L |  |  |  |  |
| Faf | Fas-associated factor | CG10372 | 2L |  |  |  |  |
| ssp3 | short spindle 3 | CG18397 | 2L |  | mitotic spindle elongation |  |  |
| CG10561 |  | CG10561 | 2L | oxidoreductase activity |  |  |  |
| pigeon | pigeon | CG10739 | 2L |  |  |  |  |
| Pax | Paxillin | CG31794 | 2L | zinc ion binding | leg disc development; regulation of Rho GTPase activity; wing disc development |  |  |
| lectin-37Db | lectin-37Dd | CG33533 | 2L | binding |  |  |  |
| CG13079 |  | CG13079 | 2L | endopeptidase inhibitor activity |  |  |  |
| sick | sickie | CG42589 | 2L | ATP binding; nucleoside-triphosphatase activity |  |  |  |
| TotF | Turandot F | CG31691 | 2L |  | Innate immune response; response to UV; response to bacterium; response to heat |  |  |
| Victoria | Victoria | CG33117 | 2L |  | Innate immune response; response to heat |  |  |
| sNPF | short neuropeptide F precursor | CG13968 | 2L | Neuropeptide hormone activity | adult feeding behavior; determination of adult lifespan; larval feeding behavior; multicellular organism growth; neuropeptide signaling pathway; positive regulation of ERK1 and ERK2 cascade; positive regulation of cell size; positive regulation of insulin receptor signaling pathway; positive regluation of multicellular organism growth; regulation of glucose metabolic process; regulation of response to food; regulation of trehalose metabolic process |  |  |
| CG9270 |  | CG9270 | 2L | ATP binding; xenobiotic-transporting ATPase activity |  |  |  |
| CG8665 |  | CG8665 | 2L | acyl carrier activity; cofactor binding; formyltetrahydrofolate dehydrogenase activity; hydroxymethyl-, formyl- and related transferase activity; methyltransferase activity; oxidoreductase activity, acting on the aldehyde or oxo group of donors, NAD or NADP as acceptor | 10-formyltetrahedrofolate catabolic process; biosynthetic process |  |  |
| Cyp6w1 | Cyp6w1 | CG8345 | 2R | electron carrier activity; heme binding; monooxygenase activity; oxidoreductase activity, acting on paired donors, with incorporation or reduction of molecular oxygen |  |  |  |
| CG2183 |  | CG2183 | 2R |  |  |  |  |
| Cyp6a13 | Cyp6a13 | CG2397 | 2R | electron carrier activity; heme binding; monoxygenase activity; oxidoreductase activity, acting on paried donors, with incorporation or reduction of molecular oxygen |  |  |  |
| Rme-8 | Receptor mediated endocytosis 8 | CG8014 | 2R | heat shock protein binding; receptor activity | border follicle cell migration; receptor-mediated endocytosis |  |  |
| l(2)03659 | lethal (2) 03659 | CG8799 | 2R | ATP binding; ATPase activity, coupled to transmembrane movement of substances |  |  |  |
| ced-6 | ced-6 | CG11804 | 2R | protein binding | apoptotic cell clearance; mushroom body development; neuron remodeling |  |  |
| dgo | diego | CG12342 | 2R | protein binding | establishment of imaginal disc-derived wing hair orientation; establishment of ommatidial planar polarity |  |  |
| CG13185 |  | CG13185 | 2R | ATP binding; ATPase activity |  |  |  |
| CG13189 |  | CG13189 | 2R | metal ion transmembrane transporter activity |  |  |  |
| MCPH1 | Microcephalin | CG42572 | 2R |  | embryo development; mitosis; mushroom body development; pole cell formation |  |  |
| CG13157 |  | CG13157 | 2R |  |  |  |  |
| CG8834 |  | CG8834 | 2R | 4-coumarate-CoA ligase activity |  |  |  |
| CG4714 |  | CG4714 | 2R |  |  |  |  |
| CG10799 |  | CG10799 | 2R |  |  |  |  |
| CG17047 |  | CG17047 | 2R |  |  |  |  |
| shot | short stop | CG18076 | 2R | actin binding; calcium ion binding; microtubule binding | actin cytoskeleton organization; apposition of dorsal and ventral imaginal disc-derived wing surfaces; axon midline choice point recognition; branch fusion, open tracheal system; cell cycle arrest; dentrite morphogenesis; determination of muscle attachment site; lumen formation, open tracheal system; microtubule cytoskeleton organization; mushroom body development; negative regulation of microtubule depolymerization; oocyte fate determination; regulation of axon extensionl sensory organ development |  |  |
| Su(var)2-HP2 | Su(var)2-HP2 | CG12864 | 2R | DNA binding | gene silencing; heterochromatin formation; mitotic chromosome condensation |  |  |
| dup | double parked | CG8171 | 2R | protein binding | DNA endoreduplication; DNA replication checkpoint; antimicrobial humoral response; centrosome duplication; cytokinesis; eggshell chorion gene amplification; mitotic sister chromatid separation |  |  |
| GaINAc-T1 | GaINAc-T1 | CG8182 | 2R | polypeptide N-acetylgalactosaminyltransferaase activity; sugar binding | oligosaccharide biosynthetic process |  |  |
| CG4282 |  | CG4282 | 2R | DNA binding; zinc ion binding |  |  |  |
| CG7813 |  | CG7813 | 2R | hydrogen-exporting ATPase activiy, phosphorylative mechanism | ATP synthesis coupled proton transport |  |  |
| Alk | Alk | CG8250 | 2R | ATP binding; transmembrane receptor protein tyrosine kinase activity | activation of MAPKK activity; axon guidance; compound eye photoreceptor development; digestive tract development; mesoderm development; regulation of hemocyte differentiation; transmembrane receptor protein tyrosine kinase signaling pathway; visceral muscle development |  |  |
| veg | vegetable | CG6657 | 2R | mannosyltransferase activity | GPI anchor biosynthetic process; peripheral nervous system development |  |  |
| CG43328 |  | CG43328 | 2R |  |  |  |  |
| CG43327 |  | CG43327 | 2R |  |  |  |  |
| CG34386 |  | CG34386 | 2R |  |  |  |  |
| CG5189 |  | CG5189 | 2R |  | cell growth; cellular protein localization; cellular response to amino acid stimulus; positive regulation of TOR signaling cascade |  |  |
| CG30120 |  | CG30120 | 2R |  |  |  |  |
| Prp19 | Prp19 | CG5519 | 2R | ubiquitin-protein ligase activity | nuclear mRNA splicing, via spliceosome |  |  |
| hts | hu li tai shao | CG43443 | 2R | actin binding; metal ion binding | cell differentiation; multicellular organismal development; oogenesis | guides photoreceptor axons | Ohler *et al.*, 2011 |
| CG11018 |  | CG11018 | 2R |  |  |  |  |
| hrg | hiiragi | CG9854 | 2R | RNA binding; polynucleotide adenylyltransferase activity | RNA polyadenylation; transcription, DNA-dependent |  |  |
| RIC-3 |  | CG30296 | 2R | receptor activity |  |  |  |
| shg | shotgun | CG3722 | 2R | beta-catenin binding; calcium ion binding | apical protein localization; axon guidance; border follicle cell migration; brain development; branch fusion, open tracheal system; cell projection assembly; gastrulation involving germ band extension; germ-line stem cell division; germarium-derived female germ-line cyst encapsulation; gonad morphogenesis; gonadal mesoderm development; head involution; homophilic cell adhesion; maintenance of epithelial integrity, open tracheal system; ommatidial rotation; oocyte anterior/posterior axis specification; oocyte localization involved in germarium-derived egg chamber formation; optic lobe placode development; outflow tract morphogenesis; pole cell migration; salivary gland morphogenesis; somatic stem cell division; somatic stem cell maintenance; wound healing; zonula adherens assembly |  |  |
| CG10527 |  | CG10527 | 2R |  |  |  |  |
| CG4030 |  | CG4030 | 2R | zinc ion binding |  |  |  |
| CG34396 |  | CG34396 | 2R | potassium channel activity |  |  |  |
| king-tubby | king tubby | CG9398 | 2R |  |  |  |  |
| CG9394 |  | CG9394 | 2R | carbohydrate binding; glycerophosphodiester phosphodiesterase activity | glycerol metabolic process; lipid metabolic process |  |  |
| Magi | Magi | CG30388 | 2R | Ral GTPase binding; guanylate kinase activity |  |  |  |
| CG30389 |  | CG30389 | 2R |  |  |  |  |
| tud | tudor | CG9450 | 2R | nucleic acid binding | P granule organization; intracellular mRNA localization; mitochondrial rRNA export from mitochondrian; pole cell development |  |  |
| CG10505 |  | CG10505 | 2R | ATP binding; ATPase activity, coupled to transmembrane movement of substances | Response to copper ion; Response to zinc ion | metal ion binding | Yepiskoposyan *et al.*, 2006 |
| CG4554 |  | CG4554 | 2R | binding |  | neurogenesis | Neumüller *et al.*, 2011 |
| CG4610 |  | CG4610 | 2R | flavin adenine dinucleotide binding | tRNA wobble uridine modification |  |  |
| CG13531 |  | CG13531 | 2R |  |  |  |  |
| CG3499 |  | CG3499 | 2R | ATP binding; metalloendopeptidase activity; nucleoside-triphosphatase activity | protein catabolic process; proteolysis |  |  |
| CG4563 |  | CG4563 | 2R | 4-coumarate-CoA ligase activity |  |  |  |
| αTub84D | α-Tubulin at 84D | CG2512 | 3R | GTP binding; GTPase activity; protein binding; structural molecule activity | cytokinesis; microtubule-based movement; protein polymerization |  |  |
| α-Est3 | α-Esterase-3 | CG1257 | 3R | carboxylesterase activity; methyl indole-3-acetate esterase activity; methyl jasmonate esterase activity; methyl salicylate esterase activity |  |  |  |
| α-Est1 | α-Esterase-1 | CG1031 | 3R | carboxylesterase activity; methyl indole-3-acetate esterase activity; methyl jasmonate esterase activity; methyl salicylate esterase activity |  |  |  |
| CG9297 |  | CG9297 | 3R | GTP binding; GTPase activity |  |  |  |
| CG15523 |  | CG15523 | 3R |  |  |  |  |
| CG15548 |  | CG15548 | 3R |  |  |  |  |
| Cyp4e1 | Cytochrome P450-4e1 | CG2062 | X | Electron carrier activity; heme binding; monooxygenase activity; oxidoreductase activity, acting on paired donors, with incorporation or reduction of molecular oxygen |  |  |  |
| CG32699 |  | CG32699 | X | 1-acylglycerophosphocholine O-acyltransferase activity; calcium ion binding | phospholipid biosynthetic process |  |  |
| CG42258 |  | CG42258 | X |  |  |  |  |
| Flo-2 | flotillin 2 | CG32593 | X | structural molecule activity | cell adhesion |  |  |
| CG12539 |  | CG12539 | X | choline dehydrogenase activity; flavin adenine dinucleotide binding; glucose dehydrogenase activity | alcohol metabolic process |  |  |
| drd | drop dead | CG33968 | X | transferase activity, transferring acyl groups other than amino-acyl groups | chitin-based cuticle development; defecation; digestive system process; oogenesis |  |  |
| RhoGEF2 | RhoGEF2 | CG9634 | X | metalloendopeptidase activity | proteolysis |  |  |
| CG6867 |  | CG6867 | X |  |  |  |  |
| Sh | Shaker | CG12348 | X | voltage-gated potassium channel activity | axon extension; behavioral response to ether; courtship behavior; detection of visible light; flight behavior; larval locomotory behavior; learning or memory; proboscis extension reflex; regulation of action potential; regulation of circadian sleep/wake cycle, sleep; regulation of synaptic activity; sensory perception of taste; sleep |  |  |
| Dhc16F | Dynein heavy chain at 16F | CG7092 | X | ATP binding; ATPase activity; microtubule motor activity; transcription factor binding | microtubule-based movement; regulation of transcription, DNA-dependent |  |  |
| CG14219 |  | CG14219 | X | transferase activity, transferring acyl groups other than amino-acyl gropus |  |  |  |
| CG11227 |  | CG11227 | X |  |  |  |  |
| Npc1b | Niemann-Pick type c-1b | CG12092 | X | hedgehog receptor activity | central nervous system development; dorsal closure; intestinal cholesterol absorption; peripheral nervous system development |  |  |
